# A novel microRNA, hsa-miR-6852 differentially regulated by Interleukin-27 induces necrosis in cervical cancer cells by downregulating the FoxM1 expression

**DOI:** 10.1038/s41598-018-19259-4

**Published:** 2018-01-17

**Authors:** Deepak Poudyal, Andrew Herman, Joseph W. Adelsberger, Jun Yang, Xiaojun Hu, Qian Chen, Marjorie Bosche, Brad T. Sherman, Tomozumi Imamichi

**Affiliations:** 1 Laboratory of Human Retrovirology and Immunoinformatics, Leidos Biomedical Research Inc., Frederick National Laboratory for Cancer Research, Frederick, Maryland 21702 USA; 2 AIDS Monitoring Laboratory, Leidos Biomedical Research Inc., Frederick National Laboratory for Cancer Research, Frederick, Maryland 21702 USA

## Abstract

We have previously demonstrated that Interleukin-27 differentially regulates the expression of seven novel microRNAs. Here we elucidate the functional significance of these novel microRNAs. Of the seven microRNAs, over expression of miRNA-6852 (miR-SX4) mimic induces cell cycle arrest at G2/M phase and induces necrosis in HEK293 and panel of cervical cancer cells (Human Papilloma Virus (HPV) infected cell lines; HeLa, CaSki and SiHa cells). To define the mechanism of the miR-SX4-mediated G2/M arrest, a microarray gene chip array and western blot analysis were performed. FoxM1, a transcription factor is identified as a key protein down-regulated by miR-SX4, even though the miR-SX4 does not target 3’UTR of FoxM1. Knock down of FoxM1 using si-RNA demonstrate that FoxM1 silenced cell induces G2/M cell cycle arrest and necrosis. Our data demonstrated for the first time that miR-SX4 could be a potent anti-cancer microRNA.

## Introduction

MicroRNAs (miRNAs) are small non-coding RNAs of 19–24 nucleotides (nts) length that post-transcriptionally regulates eukaryotic gene expression. In miRNA duplexes, the strand with the weakest 5′-end base pairing is selected as the mature miRNA and loaded onto an Argonaute (Ago) protein, whereas the miRNA* strand (passenger strand) is degraded^1^. In animals, miRNAs target transcripts through imperfect base pairing of 2–7 nts of 5′-end of miRNA (‘seed’ sequence) to multiple sites in 3′-untranslated regions (UTRs) of target mRNA, and this imperfect miRNA-mRNA hybrids with central bulges (nt 9–12) recruits miRNP (microRNA Ribonucleoprotein complex) that enable translational inhibition or exonucleolytic mRNA decay [Reviewed^2^]. Ever since its first discovery in 1993^3^, there are reports of ever-growing numbers of new microRNAs and the latest Sanger miRNA database (miRbase.org) has reported 2588 mature human miRNAs. MiRNAs play important roles in many biological processes including cell growth, apoptosis, and gene regulation, and are involved in human diseases such as cancer, vascular disease, immune disease, and infections.

The hallmarks of cancer include sustaining proliferative signaling, evading growth suppressors, resisting cell death, enabling replicative immortality, inducing angiogenesis, and activating invasion and metastasis^4^. During the neoplastic transformation, cells acquire the ability to sustain proliferation and resist cellular death or apoptosis. It is therefore essential to inhibit cell growth and induce apoptosis/necrosis in the neoplastic cells and failure to comply correctly with this cell cycle events leads to abnormalities in cell growth and function. Cancer cells often tend to forgo the cell cycle check points leading to rapid cell division resulting in a tumor mass. Progression through cell division cycle requires the periodic expression of cluster of genes that regulates the cell cycle check point (G1 and G2). By comparing the conserved complementarity of seed sequence to the target mRNA, it is estimated that 30% of all human genes are regulated by miRNA with an average of 200 target mRNAs per miRNA molecule^5^. Several miRNAs have been reported to target the mRNA that are involved in cell division cycle and cellular death^6–10^ and are often referred to as tumor suppressor miRNAs.

FoxM1 is a Forkhead box (Fox) superfamily of transcription factors which is widely expressed in proliferating cells and cancer cells. FoxM1 is a proliferation specific transcription factor and is considered as the master regulator of cell cycle as it controls the genes involved in G1/S^11^ and G2/M phase progression^12–14^ and the loss of FoxM1 generates mitotic spindle defects^15^. Given the role of FoxM1 in the progression of cell division cycle, it is also overexpressed in majority of cancer patients^16–18^, making it an important prognostic molecular marker and therapeutic target for several cancer types. Recent evidences have suggested that FoxM1 could be targeted by several tumor suppressor miRNAs^19–22^. The canonical MAPK (Mitogen Activated Protein Kinase) pathway is an upstream regulators of Fox family of proteins^23,24^. The third member of canonical MAPK pathway, ERK (Extracellular Signal-Regulated kinases) is activated through different pathways leading to different cellular responses including cellular proliferation, differentiation and survival^25,26^. Recent evidences of DNA damage leading to constitutive activation of ERK mediating cellular apoptosis are also reported^27,28^.

We originally identified Interleukin-27 (IL-27) as an anti-HIV cytokine in culture media of cervical cancer vaccine-treated cells^29^. We have previously reported IL-27 differentiates monocytes to HIV-1, HIV-2, HSV-2, Influenza and SIV resistance macrophages (I-Mac)^30^. To define the anti-viral effect, we investigated microRNA expression profile in I-Mac, and we discovered seven novel microRNAs, which are hsa-miR-7704 (-SX1), -7705 (-SX2), -7702 (-SX3), -6852 (-SX4), -SX5, -7703 (-SX6) and -7706 (-SX7)^31^. Some of these miR, -SX1, -SX5, -SX6 and –SX7 potentially targets the ORF (Open Reading Frame) of gene of HSV1, Poliovirus, HTLV4, HSV2/4, and HHV4/8^31^.

In the current study, we investigated the phenotypic and functional aspects of the novel miRNAs by determining the cell division cycle profile and cellular apoptosis/necrosis using cervical cancer cell models. Using the gene microarray and RNAi mediated silencing approach; we have also identified a key molecular target of hsa-miR-6852 (miR-SX4) as FoxM1 that is involved in mediating the anti-cancer effects of miR-SX4. Our data have future implications of these miRNAs as an attractive candidate in miRNA-based cancer therapeutics.

## Results

### miRNA-6852 (SX4) induces G2/M cell cycle arrest

**Figure 1:**
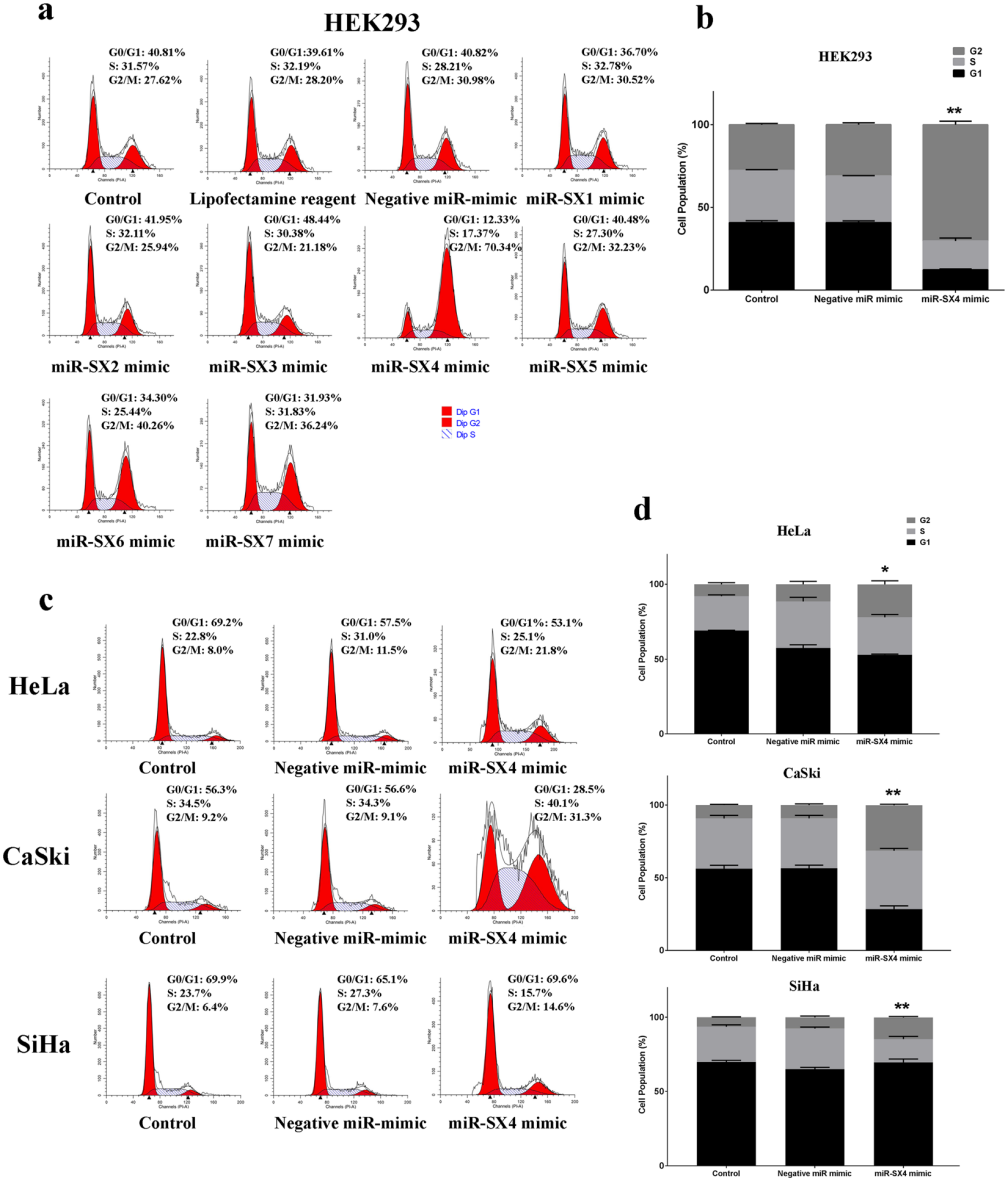
miR-SX4 induces cell cycle arrest in HEK293 cells and panel of cervical cancer cell lines. **(a**) Cell cycle phases were analyzed based on the DNA content from the histogram plot in the HEK293 cells transfected with 10 nM miRNA mimics and BD FACSDiva software was used to analyze G1,(2n) S(4n) and G2 (2n + 2n) phase of the cells, 48 h post transfection. Negative miRNA mimic was used as a negative control. X-axis indicates the PI staining (representative of DNA content) and Y-axis indicates the cell number. A minimum of 20,000 events was counted by Flow cytometry for each treatment. Representative figure is from 3 separate experiments. (**b**) Representative bar graph was plotted to show G2/M phase arrest in miR-SX4 mimic transfected HEK293 cells. (n = 3, Mean ± S.E.M; **p < 0.01). (**c**) HeLa, CaSki and SiHa cells were transfected with 20 nM of either negative miRNA mimic or miRNA-SX4 mimic and harvested 48 h post transfection for cell cycle analysis. Representative figure is from 3 separate experiments. (**d**) Representative bar graph was plotted to show G2/M phase arrest in miR-SX4 mimic transfected HeLa, CaSki and SiHa cells. (n = 3, Mean ± S.E.M; *p < 0.05, **p < 0.01).

In our previous work, we compared miRNA expression profiles between macrophage-colony stimulating factor (M-CSF) induced macrophages (M-Mac) and M-CSF with Interleukin-27(IL-27)-induced macrophages (I-Mac), and have identified seven novel miRNAs: four novel miRNAs; hsa-miR-SX1, -SX2, -SX3 and –SX6 were differentially expressed in I-Mac and three microRNAs namely SX4 (GenBank accession # KC832803 hsa-miR-6852-5p), miR-SX5 and miR-SX7 were endogenously detected in both M-Mac and I-Mac^31^, however function of each miRNA yet remains uncharacterized. To explore the phenotype of each novel miRNAs, we performed the cell cycle profiling using HEK293 and HeLa cells by transfection of the seven novel miRNA mimics, 48 hours post transfection, the cells were harvested for cell cycle analysis. Of the seven novel miRNAs, miR-SX4 mimic significantly induced cell cycle arrest at G2/M phase in HEK293 cells (Fig. 1a,b) and HeLa cells (Fig. 1c; Supplementary Fig. S1a), compared to control negative miRNA mimic (the negative miRNA mimic which is a non-targeting miRNA) transfected cells. G2/M cell cycle was increased by 2.3-fold (n = 3, p < 0.01) in HEK293; 1.9-fold in HeLa (n = 3, p < 0.05); 3.4-fold in CaSki (n = 3, p < 0.01) and 1.9-fold in SiHa cells (n = 3, p < 0.01) (Fig. 1c,d).

To confirm transfection efficiency, each miRNA level was quantified by qRT-PCR. 10 nM of each miRNA mimic transfections were confirmed in HEK293 cells (Supplementary Fig. S1b), thus, of the seven novel miRNAs, miR-SX4 specifically induces a phenotype with G2/M cell cycle arrest in HEK293 and a panel of cervical cancer cell lines.

### miRNA-6852 (SX4) downregulates FoxM1 expression

**Figure 2:**
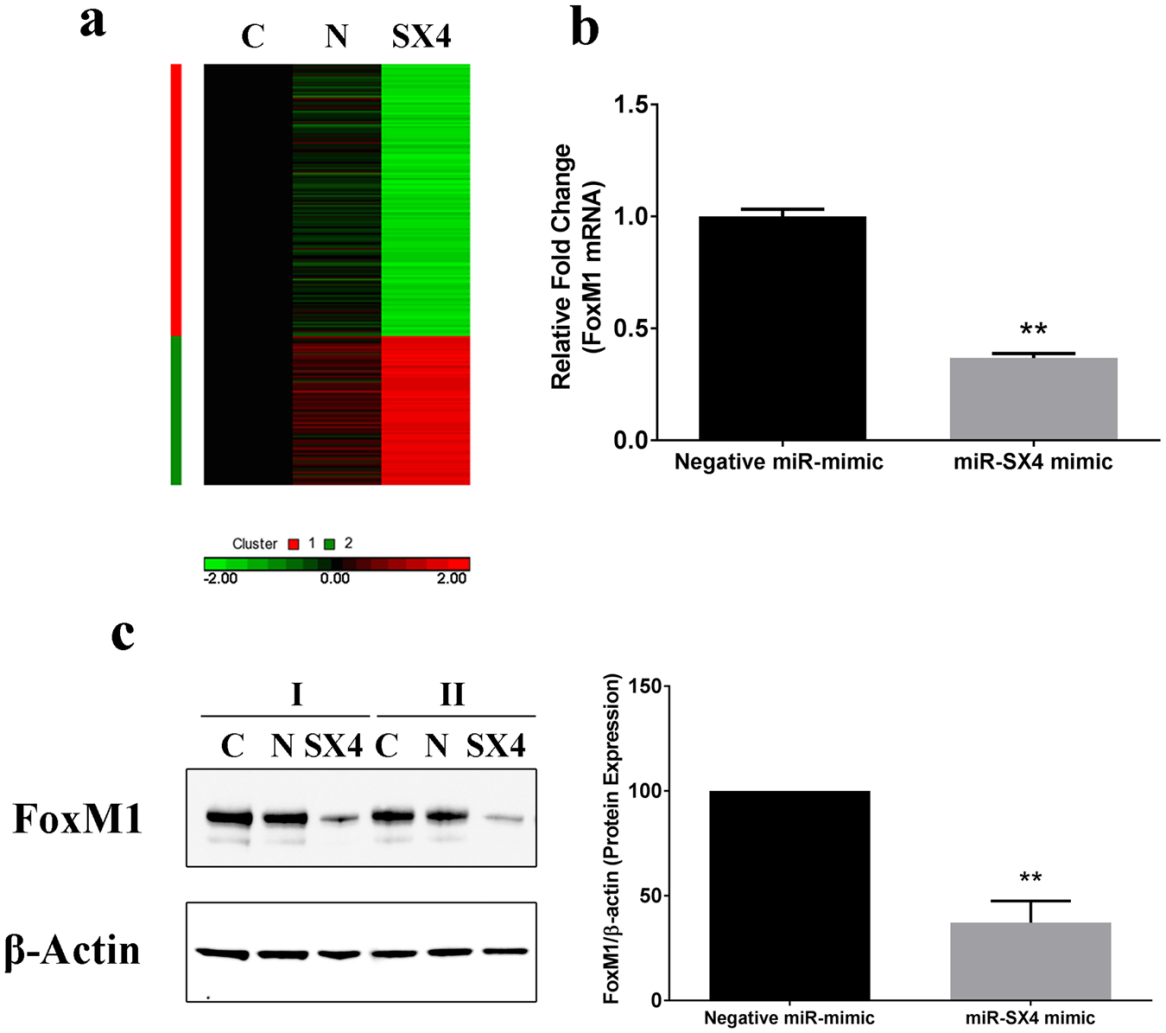
Mimic miR-SX4 downregulates FoxM1 expression (**a**) Genechip Heat Map to compare the gene expression levels in miR-SX4 mimic transfected HEK293 cells. Gene expression levels are depicted as color variation from red (high expression) to green (low expression) with fold change greater than 2 when compared with negative miR mimic control. The color in each cell of the figure displays the level of expression for each gene. C, N and SX4 represents the untransfected control, Negative miRNA mimic and mimic miR-SX4 transfected cell respectively. C, N and SX4 heat map are representative mean figure of 3 separate experiments. (**b**) FoxM1 gene expression level of miR-SX4 mimic transfected cells was quantified by RT-PCR using a gene specific probe. Data shown represents means ± SE of 3 independent studies (**p < 0.01). (**c**) The whole cell lysates of miR-SX4 transfected cells was used to determine the protein expression levels of FoxM1 and internal control β-actin by western blot analysis. The same western blot membrane was stripped and probed with β-actin antibody as a control. The protein expression of FoxM1 was quantified and normalized with β-actin expression level using NIH Image J analysis software. Data represents (average ± S.D; **p < 0.01) the normalized FoxM1 levels of miRNA-SX4 transfected cells compared to negative miRNA mimic transfected cells.

To define the key components involved in the miR-SX4 mediated cell cycle arrest, we performed a microarray gene chip assay among three sets of HEK293 cells, un-transfected cells, negative miRNA mimic and miR-SX4 transfected HEK293 cells. The heat map (baseline cutoff of ±2.0 fold change) indicates that there are 304 genes regulated by miR-SX4 mimic when compared to negative miRNA mimic control (Fig. 2a). Of these 304 genes regulated by miR-SX4, 77 genes were upregulated and 227 genes were downregulated (Supplementary Table T1). The total list of all the genes with the detailed information about the fold change are listed in Supplementary Table T1. Only 4 genes were upregulated more than 3-fold whereas 32 genes were downregulated more than 3-fold by miR-SX4 (Supplementary Table T1). To identify the genes that are involved in miR-SX4 mediated cell cycle arrest, we first investigated the genes that are downregulated by miR-SX4 and its potential target based on the target prediction database (TargetScan^32–35^) (Supplementary Table T2). There are 6 genes which are down-regulated more than 3-fold and have 3’UTR region that are potential target for miR-SX4 (Supplementary Tables T1 and T2). These are ITGA5 (−3.0 fold), NACC1 (−3.1 fold), SUSD5 (−3.2 fold), CDK6 (−3.5 fold), IMPDH1 (−3.7 fold) and CAPNS1 (−6.7 fold). None of these genes are reported as genes associated with cell cycle arrest at G2/M phase. Hence, we utilized the siRNA approach to silence the expression of these downregulated genes and accessed its effect on the cell cycle profile. The siRNA transfection induced down regulation of intended gene and protein level, but it is evident that these genes downregulation did not induce the G2/M phase cell cycle arrest (Supplementary Fig. S2a), it was speculated that a combination of multiple genes may induce the arrest. Thus we used a combination of six siRNAs at once in transfection, and analyzed the effect on cell cycle arrest, however, no significant change in cell cycle arrest was observed (Supplementary Fig. S2a,b,c) indicating that these genes are not involved in the G2/M mediated cell cycle arrest.

Since transcription factors (TFs) are involved in regulation or transcription of multiple genes involved in cell cycle^36^, we looked at the list of transcription factors that were down-regulated by miR-SX4 in the list. There is a strong possibility of these transcription factors playing an important role in the cell proliferation and growth. According to the Human transcription factor database (ATFDB 2.0. http://www.bioguo.org/AnimalTFDB/index.php) which contains 1469 human TFs, miR-SX4 upregulated 10 transcription factors and downregulated 11 transcription factors (Table 1, fold change > 2). It was well established that FoxM1 is essential for transcription of mitotic regulatory genes^37^, and there are reports that silencing of FoxM1 leads to G2/M cell cycle arrest in some cell types^12,37^. Hence, we checked the FoxM1 expression in miR-SX4 transfected HEK293 cells. Real time PCR assay and western blot assay demonstrated that overexpression of miR-SX4 significantly down-regulated FoxM1 mRNA and the protein levels by 62 ± 0.02% (n = 3, p < 0.01) and 62.3 ± 4.26% (n = 3, p < 0.01), respectively, compared to negative miRNA mimic transfected cells (Fig. 2b,c), indicating miR-SX4 directly or indirectly targets FoxM1.

**Table 1:** Human Transcription factors regulated by miR-SX4 in HEK293 cells.

Name^1^	Downregulated by miR-SX4^2^	p-value^3^	Name^1^	Upregulated by miR-SX4^2^	p-value^3^
B-Myb	−2	0.001539	ZNF329	2	0.000219
FOXM1	−2	0.0000018	ZNF682	2	0.00303
NFIC	−2.3	0.000055	ZNF814	2	0.00646
HNF1B	−2.3	0.00172	ZNF417	2.1	0.00433
MYC	−2.35	0.001619	ZNF680	2.1	0.01615
NR1H2	−2.4	0.00027	ZNF83	2.2	0.00596
MEOX2	−2.5	0.00072	ZNF587	2.2	0.0186
ARID3B	−2.7	0.000003	ZNF528	2.3	0.00035
NR2F6	−3	0.00082	ETV5	2.5	0.000083
HOXA1	−3	0.00093	NR4A2	2.9	0.000206
SOX3	−3.3	0.0013			

^1^Gene abbreviations: B-Myb, Myb-related protein B; FOXM1, Forkhead Box Protein M1; NFIC, Nuclear Factor I/C; NR2H2, Nuclear Receptor Subfamily 1 Group H Member 2; MEOX2, Mesenchyme Homeobox 2; ARID3B, AT-Rich Interaction Domain 3B; NR2F6, Nuclear Factor Subfamily 2 Group F Member 6; HOXA1, Homeobox A1; SOX3, SRY-Box 3; ZNF, Zinc Finger Protein; ETV5, ETS Variant 5; NR4A2, Nuclear Receptor Subfamily 4 Group A Member 2.

^2^Denotes fold change in gene expression after miR-SX4 mimic transfection compared to Negative miR-mimic transfected cells.

^3^Pvalue denotes statistically significant differences from 3 separate experiments.

### Silencing FoxM1 induces G2/M cell cycle arrest

**Figure 3:**
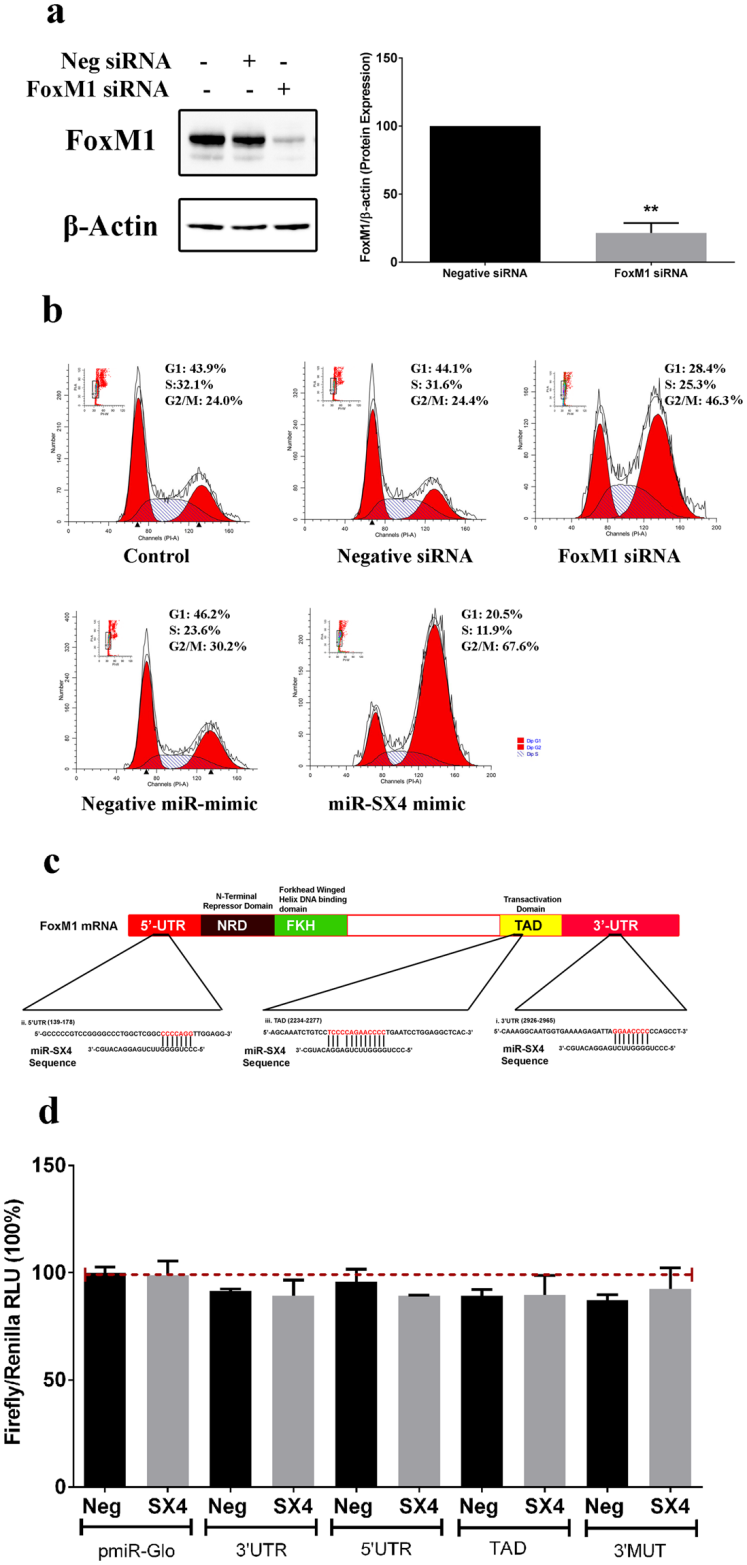
si-RNA of FoxM1 induces G2/M cell cycle arrest and miR-SX4 indirectly targets FoxM1. (**a**) 20 nM siRNA against FoxM1 and negative siRNA transfected HEK293 cells (48 h, 37 °C) were harvested and western blot analysis was performed to confirm the down-regulation of FoxM1 protein level. The same western blot membrane was stripped and probed with β-actin antibody as a control. NIH Image J was used to analyze the percentage of FoxM1/β-actin protein level down-regulated in FoxM1 siRNA transfected cells when compared to negative siRNA transfected cells. (**b**) The same set of cells transfected with si-RNA against FoxM1 and negative siRNA were harvested for cell cycle analysis. The percentage of cells in G2/M phase are displayed below the histogram plot of cell population (Y-axis) Vs PI stain (X-axis). miR-SX4 mimic, 10 nM, 48 h was used as a positive control. (**c**) Nucleotide blast was performed using NCBI nucleotide blastn program to identify the complementary sequences (>7nts) between the FoxM1 mRNA and mature miR-SX4. Three regions were identified in 5′UTR, 3′UTR and TAD region. The complementary sequence of 3′UTR region was modified and called 3′UTR MUT. These regions with complementary sequences are depicted in this figure. The oligonucleotides (60 nts length) from the identified regions were synthesized and cloned into pmiR-Glo vector. (**d**) For target validation of miR-SX4 and the regions of nucleotides in mRNA of FoxM1, the plasmid pmiR-Glo with inserted potential target sequences were cotransfected for 48 h (plasmid pmiR-Glo, 100ng and miR-SX4 (50 nM) or negative miR-mimic (50 nM)) in HEK293T cells and firefly luciferase and renilla luciferase reading was obtained. The figure represents the mean ± S.D of the Firefly/Renilla luciferase reading.

To confirm whether miR-SX4 mediated cell cycle arrest at G2/M phase is due to the downregulation of FoxM1 expression, si-RNA targeting FoxM1 was used to silence FoxM1. The siRNA downregulated FoxM1 protein by 78.6 ± 4.2% (n = 3, p < 0.01) (Fig. 3a) and increased G2/M phase by 1.9-fold (n = 3, p < 0.01), compared to negative siRNA control, G2/M arrest was similar to the mimic miRNA-SX4 [2.2 fold induced] (Fig. 3b). To further confirm the effect of FoxM1 expression in G2/M arrest in cervical cancer cells, the si-RNA mediated FoxM1 silencing in Hela cells was carried out. The silencing increased G2/M arrest by 1.45-fold (Suppl Fig. 3a).

We asked the question, if miR-SX4 directly targets the 3′UTR of FoxM1 and hence we see the observed G2/M cell cycle arrest. Using TargetScan, there was no matching sequence in the 3′UTR of FoxM1 with the seed sequence of miR-SX4. This is an indication that miR-SX4 doesn’t target FoxM1 through the conventional miRNA seed sequence and 3′UTR complementary sequence binding method. It has been reported that certain miRNA directly targets the gene when there is high complementarity between the seedless region of miRNA (the region after the ten nucleotides from the 5′end of mature miRNA comprising of at-least eight nucleotides) and the target mRNA^9^. With this in context, the complementary nucleotide sequence of entire miR-SX4 was blasted to the FoxM1 total mRNA sequence (Fig. 3c), three regions with sequence complementarity of seven nucleotide or more were potentially targeted in the mRNA of FoxM1. To define whether or not the three regions are the targets for miR- SX4, a direct target validation method using a luciferase reporter assay was performed in HEK293T cells. HEK293T cells are non-responsive to miR-SX4 and doesn’t affect the cell cycle profile (Supplementary Fig. 3b) or down regulate FoxM1 expression (Supplementary Fig. 3c). Therefore, we chose HEK293T cells for miR-SX4 direct target validation because any changes observed in the luciferase reading would be directly correlated to binding between miR-SX4 and the constructs of FoxM1. Constructs encoding luciferase gene conjugated with each three region was co-transfected with the miR-SX4 and luciferase activity was determined. The result revealed that there is no direct binding of miR-SX4 to the potential regions of FoxM1 mRNA (Fig. 3d), indicating that FoxM1 may not be the direct target of miR-SX4.

### miRNA-6852 (SX4) downregulates the transcriptional targets of FoxM1 involved in Cell cycle progression and Cell survival

**Figure 4:**
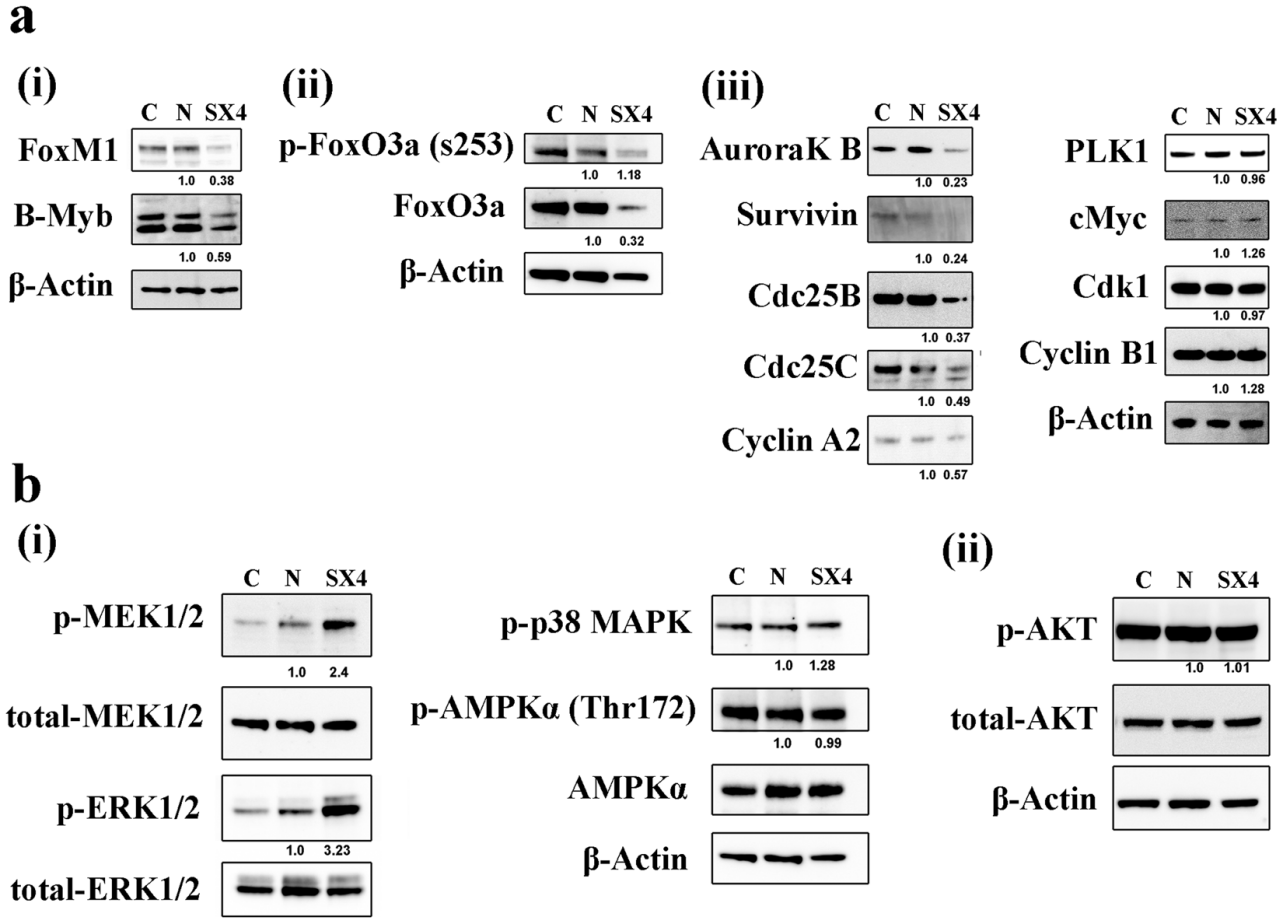
miR-SX4 reduces the expression of FoxM1 target proteins. (**a**) HEK293 cells untransfected (C), Negative miRNA mimic (N) and miRNA-6852 mimic (SX4) transfected cells were harvested and protein expression levels were determined by western blot for FoxM1, regulators of FoxM1 (FoxO3a and p-FoxO3a, B-Myb and Myc) and transcriptional targets of FoxM1 (Plk1, AuroraK B, Survivin, Cdc25B, Cdc 25 C, Cdk1, Cyclin A2 and Cyclin B1). β-actin protein expression was determined for internal control. Figure 4a: i, ii, and iii images are separate western blot gel/membranes which has been stripped and reprobed with antibodies as mentioned. (**b**) HEK293 cells: C, N and SX4 transfected cells were harvested and protein expression levels were determined by western blot for upstream regulator of FoxM1, AKT/pAKT, p-p38 MAPK, ERK/p-ERK1/2, AMPKα/p-AMPKα. β-actin protein expression was determined for internal control. Figure 4b, i and iii are same western blot gel/membranes stripped and probed with another antibody; Fig. 4b, ii is another western blot gel/membrane stripped and reprobed with antibodies as mentioned. The number below the protein band represents the densitometry analysis (NIH Image J software) of protein bands normalized to β-actin or total protein levels. Bands observed for SX4 is compared with Negative miRNA mimic control (N).

FoxM1 is reported to transcriptionally regulate several genes Plk1 (Polo Like Kinase 1), AurkB (Aurora Kinase B), Survivin (BIRC5) that are involved in G2/M transition and cell survival^37–40^. In addition, Cdc25B, Cdc25C, Cdk1, CYCLA2, and CYCLB1 are also involved in G2/M cell cycle transition^37,41–43^. Given miR-SX4 downregulates FoxM1 expression, we further investigated the effect of miR-SX4 on the expression pattern of these proteins in HEK293 cells. (Fig. 4a). The transfection down-regulated AurkB and Survivin to 23% and 24% respectively and Cdc25B, Cdc25C, and CyclinA2 that were downregulated to 37%, 49% and 57% (Fig. 4a), respectively. miR-SX4 also downregulated the protein expression of B-Myb to 59%, however, the expression of Plk1, cMyc, Cdk1 and CyclinB1 were not significantly changed (Fig. 4a). Because miR-SX4 also downregulated FoxM1 protein levels in HeLa, CaSki and SiHa cell lines, we investigated the effect of miR-SX4 in these proteins. Supplementary Figure 4a; indicated a similar pattern of protein expression in cervical cancer cell lines as observed in HEK293 cells; Additionally, the panel of cervical cancer cells also exhibited downregulation of the FoxM1 downstream targets; PLK1, Cdk1, cMyc and Cyclin B1; which remained unchanged in HEK293 cells. These results indicated that miR-SX4 transfection governs down regulation of FoxM1 and the downstream of G2/M phase regulating factors.

It is reported that the expression of FoxM1 is regulated by activation of FoxO3a^44,45^. ERK1/2^46^, p38 MAPK^47^, AKT^48^ and AMPK^44^ are also involved in FoxM1 expression. Thus, it was speculated that miR-SX4 may downregulate these regulatory factors. To define the mechanism of miR-SX4–mediated down-regulation of FoxM1, the expression of those factors were analyzed by western blot in HEK293 and panel of cervical cancer cells. In HEK293 cells, miR-SX4 transfection significantly down regulated the total amounts of FoxO3a and up-regulated ERK1/2 phosphorylation (3.2 ± 1.0 fold, n = 3, p < 0.05) and MEK1/2 phosphorylation (2.4 ± 0.5, n = 3, p < 0.05), but had no impact on phosphorylation/activation of p38MAPK, AKT and AMPK (Fig. 4b). However, miR-SX4 upregulated the phosphorylation of MEK1/2 and ERK1/2 only in CaSKi and SiHa cells; but not in HeLa cells lines. Accordingly, in all the cervical cancer cells tested, miR-SX4 had no impact on phosphorylation/activation of p38MAPK, AKT and AMPKα (Suppl Fig. 4b).

### miRNA-6852 (SX4) induces necrosis in cancer cell lines (panel of cervical cancer cells)

**Figure 5:**
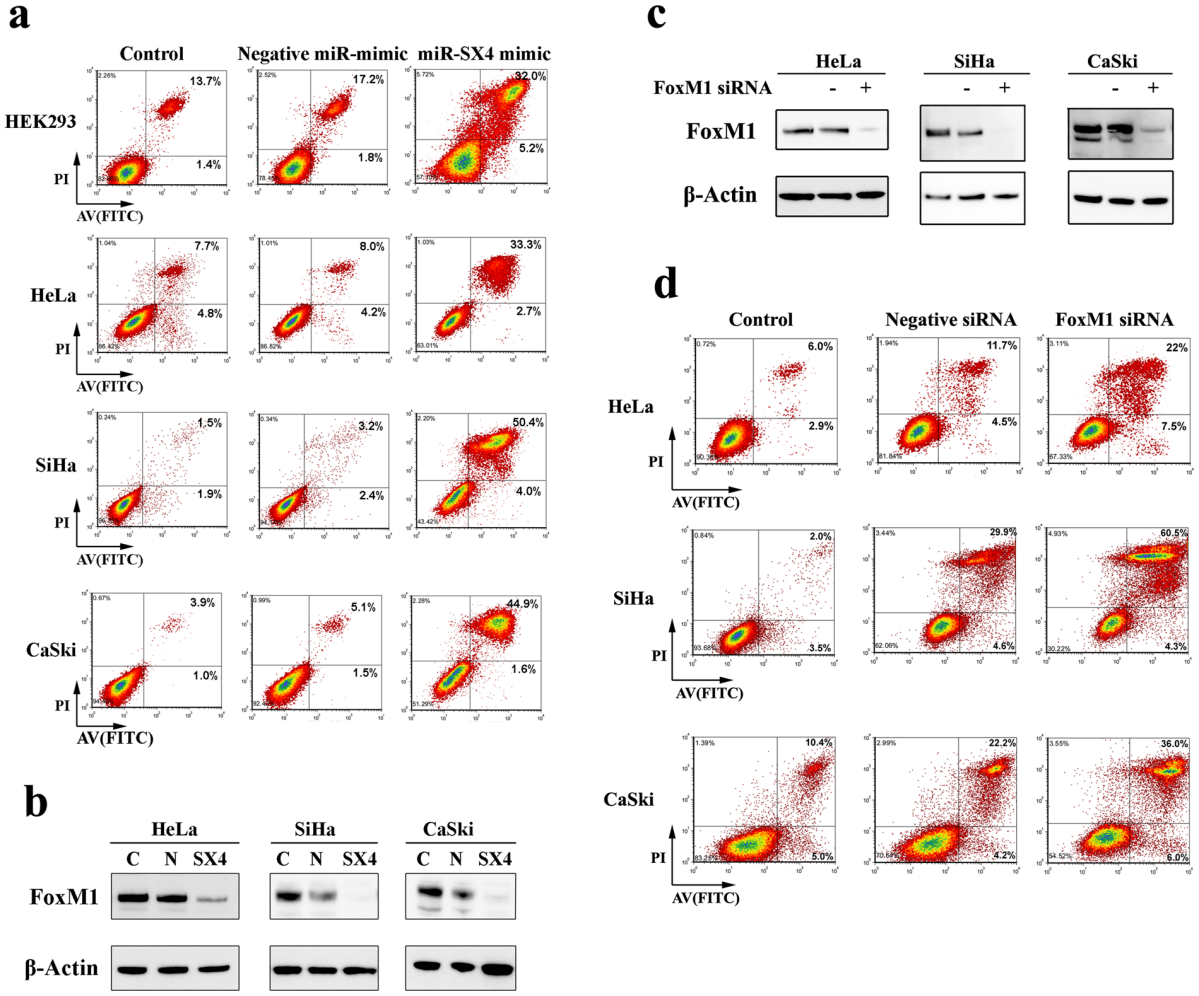
miRNA-SX4 and siRNA-FoxM1 induces necrosis in Cervical Cancer Cell lines. (**a**) Apoptosis/Necrosis analysis of HEK293 cells and cervical cancer cell lines (HeLa, SiHa and CaSki cells), 72 h post transfection of miR-SX4 mimic (20 nM), negative miR mimic was used as a negative control. Top right quadrant AV + /PI + population indicative of late apoptosis or necrotic cells are monitored. (**b**) Western blot analysis of FoxM1 protein expression in miR-SX4 transfected HeLa, CaSki and SiHa cells. Each membrane was stripped and probed with β-actin control antibody as mentioned. (**c**) Western blot analysis was performed in the same set of HeLa, CaSki and SiHa cells transfected with siRNA against FoxM1 and non-targeting negative control to determine the downregulation of FoxM1 protein level. Each membrane was stripped and probed with β-actin control antibody as mentioned. (**d**) siRNA (20 nM) against non-specific target (negative siRNA) and FoxM1 transfected HeLa, CaSKi and SiHa cells (72 h), were harvested for AV/PI stain. Apoptosis/Necrosis was analyzed using a flow cytometry and representative dot plot is shown.

Our observation have revealed that miR-SX4 downregulated the expression of several cancer cell survival proteins such as AurkB and Survivin^39,49,50^, and hence there is a potential of miR-SX4 to induce necrosis in cancer cells. miR-SX4 induced necrosis up to 2-fold (from 19% to 37.2%) in HEK293 cells when compared to its negative microRNA mimic (Fig. 5a). To further extend the necrosis-inducing profile, we evaluated the inducing function using HeLa cells. miR-SX4 was more potent in inducing necrosis in HeLa cells by 4.2-fold (from 8% to 33.3%) (Fig. 5a). Since HeLa cell line is known as human papilloma virus (HPV) transformed cell line, to define whether or not the miR-SX4 is able to induce necrosis in other HPV-infected cell line, we accessed the function using SiHa and CaSki cell lines. Necrotic stage in negative miR mimic-transfected SiHa and CaSki demonstrated 3.2 and 5.1%, respectively, and the miR-SX4 transfection induced necrosis in SiHa and Caski to 50.4% (15.8-fold) and 44.9% (8.8-fold), respectively (Fig. 5a) along with the down regulation of FoxM1 (Fig. 5b). Those data indicated that the expression of FoxM1 is involved in the cancer cell death. To further precisely demonstrate a correlation of FoxM1 expression and necrosis, FoxM1 was knocked down using siRNA. Figure 5c, indicated the efficiency of siRNA mediated silencing of FoxM1 expression at protein level. Figure 5d indicated that silencing FoxM1 expression in HeLa cells leads to 1.8-fold increase in the total necrotic cells (from 16.2% to 29.5%); in CaSki and SiHa, necrotic cells increased to 1.6 fold (26.6% to 42%) and 1.9 fold (from 34.5 to 64.8%) respectively when FoxM1 was silenced. Hence FoxM1 is also involved with the cell survival and silencing its expression leads to increased necrosis in cervical cancer cell line.

## Discussion

In the present study, we demonstrated that, miR-SX4 (official miR ID: hsa-miR-6852) overexpression leads to the G2/M phase cell cycle arrest and induces necrosis in HEK293, HeLa, SiHa and CaSKi cells. We have previously reported that seven novel miRNAs including miR-SX4 are detected in IL-27 treated macrophages^31^. To further delineate the phenotype and function of each of these seven novel miRNAs, we analyzed a cell cycle profiling after transfection of mimic miRNA in HEK293 and HeLa cells because the novel microRNAs except miR-SX5 have endogenously low levels of expression in HEK293 and HeLa cells (Supplementary Fig. S5) and the phenotypic effect observed is directly due to the exogenous mimic miRNA. This is the first time any functional and phenotypic assessment of these novel miRNAs has been conducted. Several miRNA’s that simultaneously induces cell cycle arrest and apoptosis suggesting the role of such miRNA as tumor suppressors^10,51^. Of the seven novel microRNAs, microRNA-SX4 significantly induced G2/M phase cell cycle arrest in HEK293, HeLa and SiHa cell (nearly 2-fold increase); and CaSki cells (3.4-fold increase) (Fig. 1, Supplementary Fig. S1a), indicating its potential as a tumor suppressor miRNA.

In our present study, to identify the target of miR-SX4, we performed a combination of analysis using gene expression profile, TargetScan and annotation analysis using DAVID^52,53^. Despite the fact that 6 genes were selected as potential target genes, siRNA downregulation of these genes didn’t induce cell cycle arrest. The eukaryotic transcription factors are crucial elements in regulation of the gene expression and maintenance of cellular state and function including cell cycle progression. Furthermore, altered miRNA expressions may contribute to the characteristics of cells including cancer cells [Reviewed in^54^]. Therefore, it is imperative to study the regulation of transcription factors mediated by microRNAs. Of the 21 transcription factors regulated by miR-SX4, FoxM1 have been extensively reported to be involved in cell cycle progression and in regulation of cell survival genes^12,14,15,43,55,56^; and its elevated expression is positively correlated with tumor progression and poorer prognosis of cancer^42,57,58^. Based on phenotype of cell cycle arrest, we discovered the miR-SX4 overexpression downregulates FoxM1. The FoxM1 downregulation by the siRNA induced G2/M cell arrest by approximately 1.9-fold in HEK293 (Fig. 3b); 1.45-fold in HeLa cells (Supplementary Fig. 3a); while miR-SX4 transfection induced it by 2.2-fold and 1.9-fold in HEK293 and HeLa cells respectively (Fig. 3b and Supplementary Fig. 3a), suggesting that miR-SX4 has more potent cell cycle arrest property than si-FoxM1 alone. Target scan analysis and luciferase assay demonstrated that, the miR-SX4 doesn’t directly target 3’UTR of FoxM1 or the mRNA transcripts itself (Fig. 3c,d), although we attempted to define the mechanism of downregulation by qPCR or western blot, we are not able to precisely determine the mechanism of the downregulation. However, miR-SX4 is able to downregulate the expression of B-Myb (Fig. 4a) which is known to be associated positively with FoxM1 expression^59^, and B-Myb along with MuvB is reported to recruit FoxM1 to late gene promoters during G2 phase to complete the cell division cycle^55,56^. Our observation of both B-Myb and FoxM1 downregulation by miR-SX4 and hence the G2/M cell cycle arrest could be due to the lack of recruitment of FoxM1 to the late gene promoters during G2 phase. MicroRNA-SX4 also downregulated FoxO3a which has been reported to negatively regulate FoxM1 expression^45,60^. TargetScan analysis of B-Myb and FoxO3a did not reveal any potential targets sites for miR-SX4. The siRNA against FoxM1 not only downregulated FoxM1 but also FoxO3a protein expression by 72%, and B-Myb protein expression by 80% (Supplementary Fig. S6a), indicating that the miR-SX4 mediated FoxO3a and B-Myb downregulation (Fig. 4a) is due to the FoxM1 downregulation, hence FoxM1 could potentially be acting upstream regulating FoxO3a and B-Myb in miR-SX4 overexpressed cells. There are mixed reports of FoxO3a being the antagonistic against FoxM1 transcriptional output^61^ and both FoxO3a and FoxM1 also may cooperatively regulate the gene transcription^62^. In our study, miR-SX4 could be negatively regulating FoxM1, as a result of which FoxO3a is also downregulated; and may collectively be involved in cell cycle arrest and necrosis in certain cell types (in HEK293 and CaSki cells but not in HeLa and SiHa cells, Fig. 4a and Supplementary Fig. 4a). This notion of whether FoxM1 and FoxO3a cooperatively regulates the genes required for cell cycle arrest and necrosis in cancer cells warrants further investigation. Regardless, we have evidence to prove that miR-SX4 downregulates the FoxM1 expression, which is the key mechanism of G2/M cell cycle arrest. Additionally, the B-Myb downregulation by siRNA against B-Myb had no effect on FoxM1 expression but the siRNA against FoxM1 was able to downregulate B-Myb protein expression (Supplementary Fig. S6b), indicating that B-Myb could be downstream of FoxM1 in miR-SX4 overexpressed cells.

The third member of canonical MAPK signaling, MEK/ERK is reported to mediate G2/M effect via regulating FoxM1 function^24^. Contrastingly, the phospho-MEK1/2 and phospho-ERK1/2 was also up-regulated and constitutively phosphorylated by miR-SX4 (Fig. 4b), indicating that miR-SX4 has impact on the expression of those FoxM1 regulatory proteins as an off-target effect. Furthermore, constitutively elevated ERK1/2 activities by some DNA damage are reported to exhibit apoptotic effects by mediating pro-apoptotic signaling^27,63,64^. Therefore, it cannot be ruled out that miR-SX4 induces DNA damage that constitutively activates ERK1/2 signaling to facilitate cellular death. To further determine if constitutively phosphorylated ERK1/2 leads to downregulation of FoxM1 expression, we used phospho-ERK1/2 specific inhibitor (SCH772984) in the presence of miR-SX4, and checked the protein expression level of FoxM1. miR-SX4 in the presence of SCH772984 was unable to restore the FoxM1 protein level (data not shown), indicating that even when ERK1/2 phosphorylation was inhibited, miR-SX4 was able to downregulate FoxM1 expression, thereby indicating that phosphorylated ERK-1/2 and downregulated FoxM1 expression by miR-SX4 are two separate mechanisms. Additionally, the upregulation in phosphorylation of MEK-1/2 and ERK-1/2 is cell type dependent as upregulation was observed in CaSki and SiHa cells, however downregulation was observed in HeLa cells indicating an off-target effects of miR-SX4. However this is not uncommon, given the nature of microRNA, a single miRNA targets on an average about 200 transcripts^5^ and hence an off-target effects of miRNA is well reported^65^.

The FoxM1 downregulation by the siRNA induced necrosis by approximately 1.8-fold in HeLa; 1.6-fold in CaSki and 1.9-fold in SiHa (Fig. 5c), while miR-SX4 transfection induced it by 4~16 fold, suggesting that miR-SX4 has more potent anti-cervical cancer property than si-FoxM1 alone. Because miR-SX4 doesn’t directly targets 3’UTR of FoxM1 but downregulation of FoxM1 induces G2/M phase cell cycle arrest and cellular necrosis, we performed rescue experiments by overexpressing FoxM1 in miR-SX4 transfected cells. miR-SX4 was transfected first and then FoxM1 was overexpressed in HEK293 cells, however this did not rescue the G2/M arrest and necrosis (Supplementary Fig. S7a,b). The reason for this is that miR-SX4 has a prominent effect in terms of FoxM1 downregulation and the cells are already undergoing necrosis and further overexpression of FoxM1 was not sufficient to rescue G2/M arrest or necrosis in HEK293 cells. Another possibility for not rescuing the G2/M arrest or necrosis in FoxM1 overexpressed cells is miR-SX4 also significantly downregulated even the overexpressed FoxM1 protein (Supplementary Fig. S7c) and therefore no recovery in G2/M cell cycle arrest or necrosis is observed. To overcome such dominating effect of miR-SX4 in downregulating FoxM1 expression, we increased the amount of FoxM1-GFP construct to overexpress higher amount of FoxM1 in the cells, however the higher concentration of the DNA construct (more than 1μg) was detrimental to the cell survival (data not shown). We utilized another approach of rescue effect in cervical cancer cell line, HeLa. Because in the first approach, we overexpressed miR-SX4 first followed by FoxM1 overexpression, we suspect that the necrotic effects of miR-SX4 is extremely high to overcome because most of the cells are already in the process of necrosis/cellular death (Supplementary Fig. S7a,b). Because in our first approach of FoxM1 overexpression for restoration of cell arrest or necrosis, the overexpressed FoxM1 protein level was never restored in the presence of miR-SX4, we utilized the second approach using cervical cancer cell line, HeLa, where FoxM1 was overexpressed first, followed by miR-SX4 transfection so that FoxM1 is already overexpressed in these cells before miR-SX4 is introduced which could also overcome the extremely high rate of cellular death induced by miR-SX4. Even in this approach, FoxM1 overexpression did not restore G2/M cell arrest and necrosis (Supplementary Fig. S7d,e). The reason for this is even when FoxM1 was overexpressed first followed by transfection with miR-SX4, miR-SX4 significantly downregulated the overexpressed FoxM1 protein expression (Supplementary Fig. S7f). Hence using these two approaches, we find it impossible to perform the rescue experiment correctly to confirm that FoxM1 could restore G2/M cell arrest or necrosis in cervical cancer cells. However, these observations from both the cell types (HEK293 and HeLa) concludes that even the overexpressed FoxM1 is greatly downregulated in the presence of miR-SX4 which ultimately is linked to the cell cycle arrest and necrosis thereby proving our hypothesis that downregulation of FoxM1 is key in miR-SX4 mediated cell cycle arrest and necrosis. To define sensitivity to the miR-SX4, we performed a pilot experiment to access the expression level of miR-SX4 in NCI60 panel of human tumor cell lines. Endogenous expression levels of miRNA-SX4 was compared among cells. Compared to Hela Cells, the ovarian, cervical, melanoma (exception of MDA-MB-435) and colon (exception of HT-29) cancer cells demonstrated a lower level of the miR-SX4 expression. This indicates that there is a potential of anti-cancer effect of miRNA-SX4 against these forms of cancer sub-types (Supplementary Fig. S8). Given that, miR-SX4 was first detected in IL-27 treated macrophages^31^ and we have previously reported that IL-27 exerts anti-HIV effects in Monocyte Derived Macrophages (MDM)^29,30,66^, CD4+T cells^67^ and mature DCs^68^. Therefore, there is also a potential that miR-SX4 may functionally exert anti-HIV effects in these primary target cells of HIV virus. The anti-viral study of miR-SX4 will be pursued as a separate study and will be reported separately.

In conclusion, we have determined the anti-cancer role of miRNA-6852 (miR-SX4) using different cell lines and have shown it to be particularly effective against cervical cancer cells, by significantly regulating transcription factor FoxM1. Clearly, we have demonstrated that HeLa, CaSki and SiHa cells responds to miR-SX4 mimic for cell cycle arrest and necrosis. By setting the response cutoff baseline to the endogenous expression level of HeLa cells, we have predicted that ovarian, cervical, melanoma and colon tumor cells may respond to miR-SX4 in NCI-60 panel of human tumor cell lines. Therefore miRNA-6852 (SX4) is a novel microRNA differentially regulated by IL-27, which exerts anti-cancer effects by inducing G2/M arrest and cellular necrosis by regulating FoxM1.

## Methods

### Cells and reagents

Human cervical cancer cell lines (HeLa, CaSki, SiHa), human embryonic kidney 293 (HEK293) and HEK293T cell lines were obtained from American Type Culture Collection (Rockville, MD, USA) and maintained following manufacturer’s instructions. HeLa, HEK293 and HEK293T cell lines were maintained in D-10 medium [D-MEM (Thermo Fisher Scientific) with 10% FBS, 10 mM HEPES and 5 µg/ml of Penicillin/Streptomycin]. CaSki cell line was maintained in RP-10 medium [RPMI-1640 (Thermo Fisher Scientific) with 10% FBS, 10 mM HEPES and 5 µg/ml of Penicillin/Streptomycin]. SiHa cell line was maintained in E-10 medium [E-MEM (Thermo Fisher Scientific) with 10% FBS, 10 mM HEPES and 5 µg/ml of Penicillin/Streptomycin]. NCI-60 Human Tumor Cell pellets were obtained from Developmental Therapeutics Program (DTP) of the NCI. Anti-FoxM1, Anti-Survivin, Anti-B-Myb, Anti-Myc, Anti-Cyclin B1, Anti-Cdk1 and Anti-B-Actin antibodies were purchased from Santa Cruz BioTechnology. Anti-Cdc25B was obtained from Origene. Anti-AuroraK B and Anti-Plk1 were obtained from Cell Signaling. Anti-Cyclin A1 and Anti-Cdc25c were obtained from Millipore. Si-RNA against FoxM1, B-Myb, FoxO3a and Negative control siRNA were obtained from Origene. Si-RNA against CDK6, IMPDH, ITGA5, CALPAINS1, NACC, SUSD5, NR4A2, SMPX, TMSB15A, TTC18, FAM227A, and Negative control siRNA were purchased from Thermo Fisher Scientific. miRNA mimics for mature miRNA-7704, -7705, -6852, -SX5, -7703, -7706 and control negative miRNA mimic were obtained from Thermo Fisher Scientific.

### MicroArray Analysis

DNA microarray assay was performed using the Affymetrix GeneChip System (Affymetrix). The Affymetrix Human Exon 1.0 ST Array containing 1.4 million probe sets was used. Total cellular RNA was extracted from HEK293 cells transfected with Negative miRNA mimic and mimic miRNA-6852 by using phenol-chloroform based extraction using Qiazol (Qiagen) and quantitated following the manufacturer’s protocols (Affymetrix). Terminal labeling and hybridization, array wash, stain, and scan were processed according to the Affymetrix recommended standard protocol. Intensity data were processed and summarized to gene level with Partek (Partek). Differentially expressed gene candidates were selected for verification with an absolute fold change difference >2.0.

### Transfection of miRNA mimics, siRNA silencing of genes; overexpression of FoxM1

Reverse transfection protocol was followed for si-RNA and miRNA mimics transfection. Briefly, 20 nM of si-RNA and 10 or 20 nM of miRNA mimics transfection mix was prepared in a Lipofectamine RNAiMax transfection reagent (Thermo Fisher Scientific), following the manufacturers protocol and plated on the 6 well plate first. Following this, 1.5 × 10^5^ cells were seeded in medium in the same 6 well plates. 24 h later, the medium with the transfection reagent was replaced with fresh medium. After 24 h of medium replacement for miRNA mimic and si-RNA transfection (total of 48 h) the cells were harvested for Real time RT-PCR, cell cycle analysis and western-blot analysis. After 48 h of transfection, total RNA was extracted using Qiazol regent. RNA concentration was determined by Nanodrop 1000. 100 ng of total RNA was reverse-transcribed to cDNA using TaqMan MicroRNA Reverse Transcription kit with microRNA primers specific for hsa-miR-SX1-SX7 and the small nuclear protein RNU44 (U44) for normalization. qPCR measurement of miRNAs and U44 expression was performed using TaqMan MicroRNA Assays with the CFX-96 Real Time System (BioRad). The relative fold change in miRNA level was used to represent the relative abundance of miRNAs compared with U44 expression. All experimental control samples were treated with an equal concentration of a non-targeting negative control sequence, for use as controls for non-sequence-specific effects in miRNA and siRNA experiments. Mock-transfected controls did not produce any significant effect on any of the parameters analyzed. For the overexpression of FoxM1 for rescue experiment, we utilized two approaches. The first approach was overexpression of miR-SX4 (10 nM) by reverse transfection and 24 h later, same cells were transfected with 1μg of the plasmid DNA construct (FoxM1-GFP tagged) (Origene) using TransIT-293 transfection reagent (Mirus Bio). The cells were harvested 48 h after the DNA transfection. The second approach was overexpression of plasmid DNA construct (FoxM1-GFP tagged) by reverse transfection and 24 h later, same cells were transfected with miR-SX4 or negative miR-mimic. The cells were harvested 48 h after the microRNA transfection. The cells were harvested for cell cycle analysis, apoptosis/necrosis assay were performed on GFP+cells. Western blot experiments were performed on the harvested cells.

### Quantitative RT-PCR

Cells were washed with cold PBS (Quality Biology) and total RNA was extracted using Qiazol reagent (Qiagen). One μg of total RNA served as template for single strand cDNA synthesis in a reaction using Taqman Reverse Transcription Reagents (Thermo Fisher Scientific) with random hexamer priming. Expression levels of the genes of interest were measured by semi-quantitative RT PCR by a CFX96 Real Time System (BioRad). The level of gene expression was normalized to GAPDH. Probes specific for FoxM1 and GAPDH were purchased from Thermo Fisher Scientific.

### SDS-PAGE and Western blot Analysis

Cells (1.5 × 10^5^) seeded in 6-well plate, after mimic miRNA and si-RNA transfection, 48 h at 37 °C were washed with ice-cold PBS, and resuspended in RIPA buffer (Boston Bioproduct) with protease inhibitor cocktail (Sigma Aldrich) and phosphatase inhibitors (Thermo Fisher Scientific) at 4 °C for 10 min. The protein concentration was determined using the bicinchoninic acid (BCA) protein assay kit (Thermo Fisher Scientific). Using a total of 20 μg protein, western blot analysis was performed as previously described^66^. Antibody binding was visualized using ECL Prime Western Detection Reagent (GE-Healthcare) and LAS-4000 (Fujifilm, Tokyo, Japan). The intensity of the band was analyzed by NIH ImageJ (http://rsbweb.nih.gov/ij/). The western blot membrane was stripped (Restore Western Blot Stripping Buffer, Thermo Fisher Scientific) and reprobed with another antibody or β-actin wherever necessary.

### Cell cycle analysis and Apoptosis Assay

The cells were harvested after 48 h of reverse transfection and cell cycle assay was performed by labeling the dsDNA of the cells with PI (Propidium Iodide) (Sigma). Briefly, the harvested cells were fixed in 70% ethanol for 30 minutes at 4 °C. The cells were washed in PBS and resuspended in 1 ml of PBS. 5 μl of 10 mg/ml RNase A solution (Sigma) was added to the cells and incubated at 37 °C for 15 min. 10 μl of 1 mg/ml solution of PI was then added to the cells and incubated at 4 °C for at least 30 min before being analyzed on the flowcytometer. 20,000 events were captured on the flow-cytometer (BD LSR Fortessa, BD Biosciences) Based on the DNA content, the different phases of the cell cycle was determined by using Modfit LT 3.2 cell cycle software. Cells were harvested for Annexin V (AV) according to instructions provided by the kit manufacturer (Alexa fluor 488 Annexin V/Dead cell apoptosis kit, Invitrogen. Annexin V/propidium iodide (PI) staining was examined using a flow cytometer (BD LSR Fortessa). Data was analyzed with FSCExpress V6 software. Experiments were repeated three times.

### Luciferase Reporter Assay

HEK293T (1.5 × 10^4^) cells were plated in 96-well plates 24 h prior to transfection. pmiR-Glo luciferase reporter plasmids containing 100ng of 5′UTR, 3′UTR, TAD and 3′UTR mutated regions (40 nts) were co-transfected with miRNA-6852 mimic (50 nM) or negative miR mimic control (50 nM) using Lipofectamine 2000 (Thermo Fisher Scientific). The sequence information of constructs of 5′UTR, 3′UTR and TAD region are provided in Fig. 3c. The sequence information of 3′UTR mutated region (bold) is 5′-CAAAGGCAATGGTGAAAAGAGATTA**ATATCGAG**CCAGCCT-3′. Firefly and Renilla luciferase activities were determined 48 h after transfection using the dual-luciferase reporter assay system (Promega Corporation) following the manufacturers protocol. The Renilla values were normalized to firefly luciferase.

### Statistical Analysis

Statistical analyses were performed using GraphPad Prism 5 software. Error bars indicate standard deviations (SD) or standard errors (SE) from means as noted. An unpaired Student’s test was used and p values lower than 0.05 were considered significant.

## Electronic supplementary material

Supplementary Figures

Supplementary Table T1

Supplementary Table T2
