# Supplementary material for: A novel microRNA, hsa-miR-6852 differentially regulated by Interleukin-27 induces necrosis in cervical cancer cells by downregulating the FoxM1 expression
Source: Sci Rep. 2018 Jan 17;8:900. doi: 10.1038/s41598-018-19259-4 (PMC5772045; doi:10.1038/s41598-018-19259-4)

Supplementary Figures

A novel microRNA, hsa-miR-6852 differentially regulated by Interleukin-27 induces necrosis in cervical cancer cells by downregulating the FoxM1 expression.

Deepak Poudyal, Andrew Herman, Joseph Adelsberger, Jun Yang, Xiaojun Hu, Qian Chen, Marjorie Bosche, Brad T. Sherman, and Tomozumi Imamichi

Supplementary Figure S1

Figure S1a

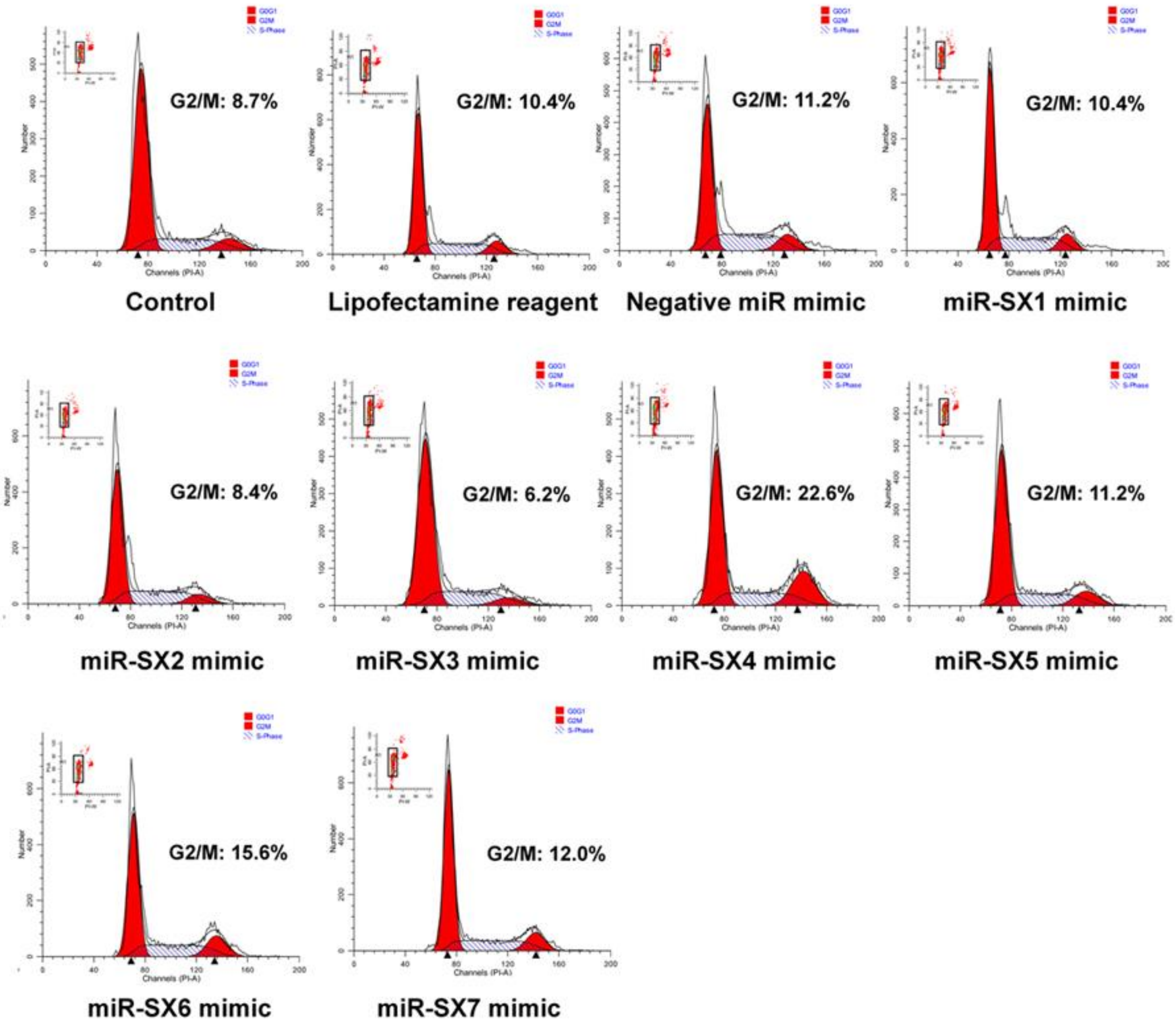

Figure S1b

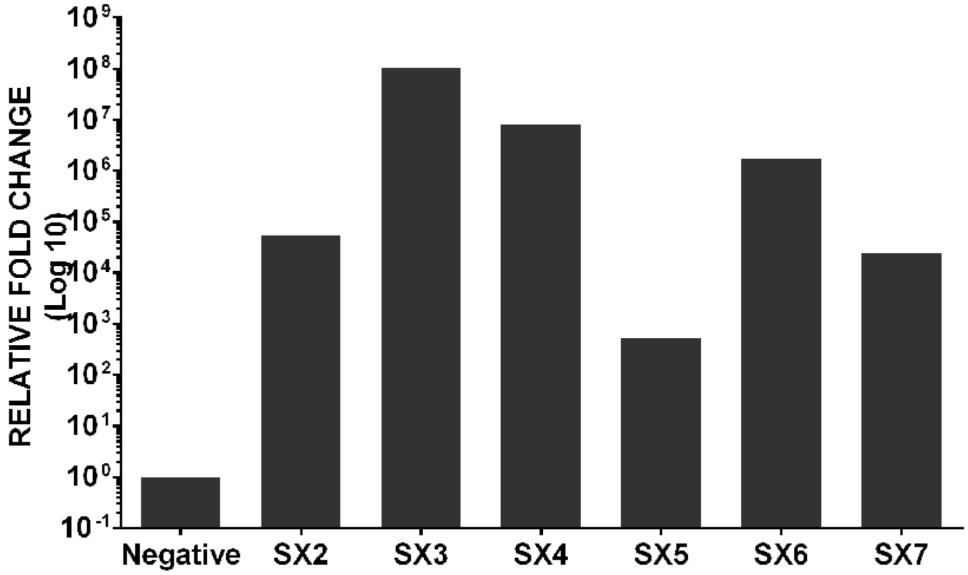

Supplementary Figure S1. miR-SX4 induces G2/M arrest. (a) Histogram plot of cell cycle analysis of HeLa cells transfected with miRNA mimic (10nM, 48h). (b) mi-RNA transfection efficiency determined by miRNA-RT-PCR analysis normalized by internal control (RNU44) in HEK293 cells. Negative bar is negative miRNA mimic transfected samples from which cDNA of 6 novel miRNAs (SX2-7) were synthesized and microRNA q-RT PCR was performed and expression of each novel miRNA for Negative miRNA mimic sample was set to 1 for relative comparison of the expression of the miRNAs.

Supplementary Figure S2

Figure S2a

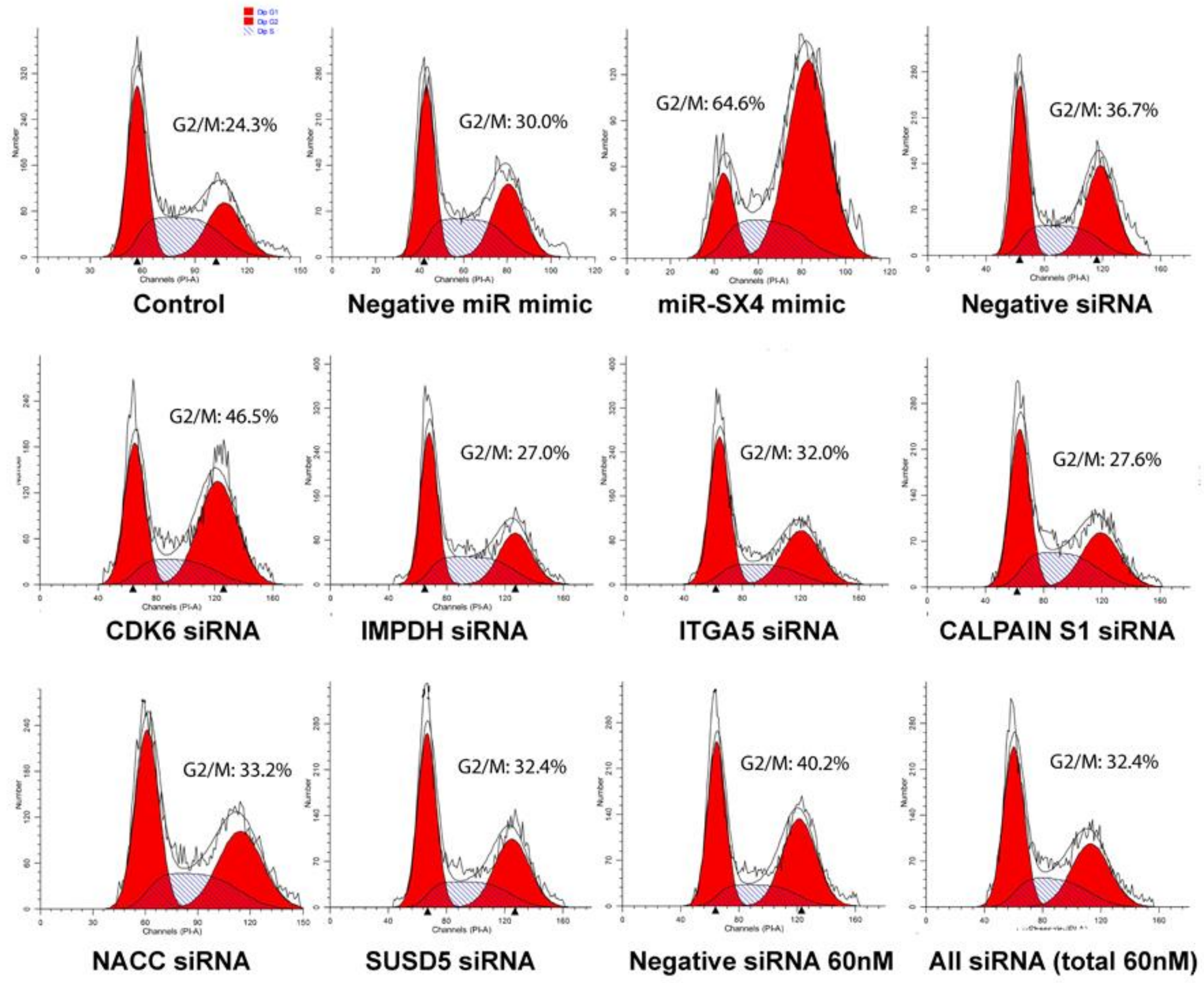

Figure S2b

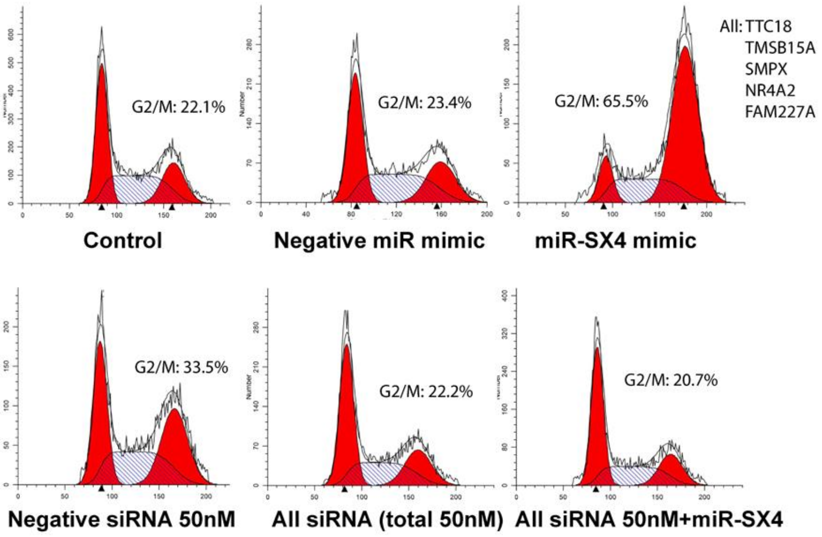

Figure S2c

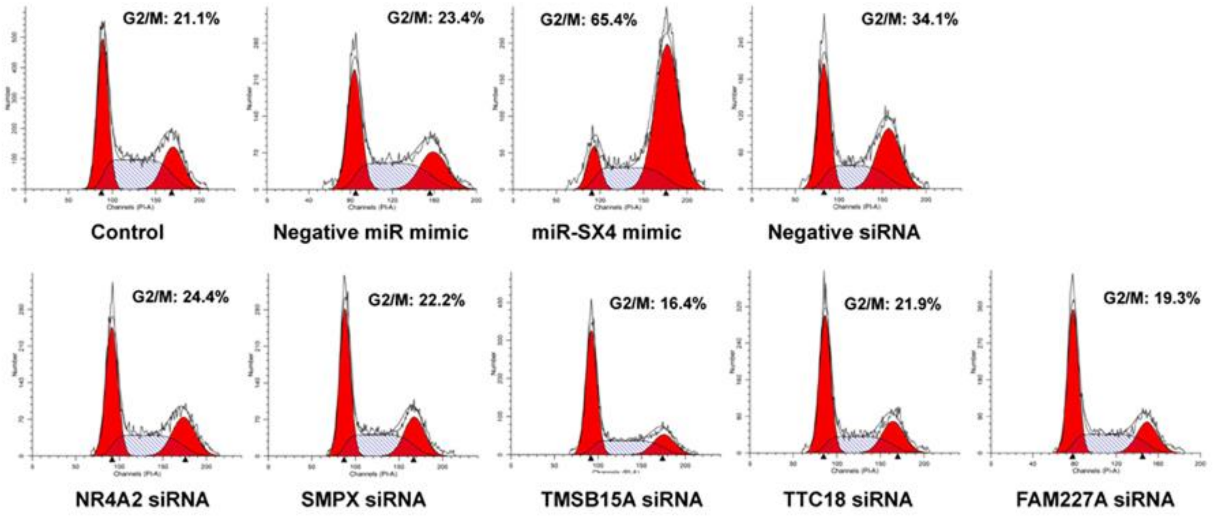

Supplementary Figure S2. (a) Histogram plot of cell cycle analysis in HEK293 cells transfected with si-RNA (10nM, 48h) of 6 genes that are down-regulated in genechip, that are also potential 3'UTR targets of miR-SX4. (b) Histogram plot of cell cycle analysis in HEK293 cells transfected with si-RNA (10nM, 48h) of the genes that are up-regulated in genechip, that are also potential 3'UTR target of miR-SX4. (c) Cell cycle analysis of si-RNA of up-regulated cells with mimic miR-SX4 for cell cycle recovery analysis.

Supplementary Figure S3

Figure S3a

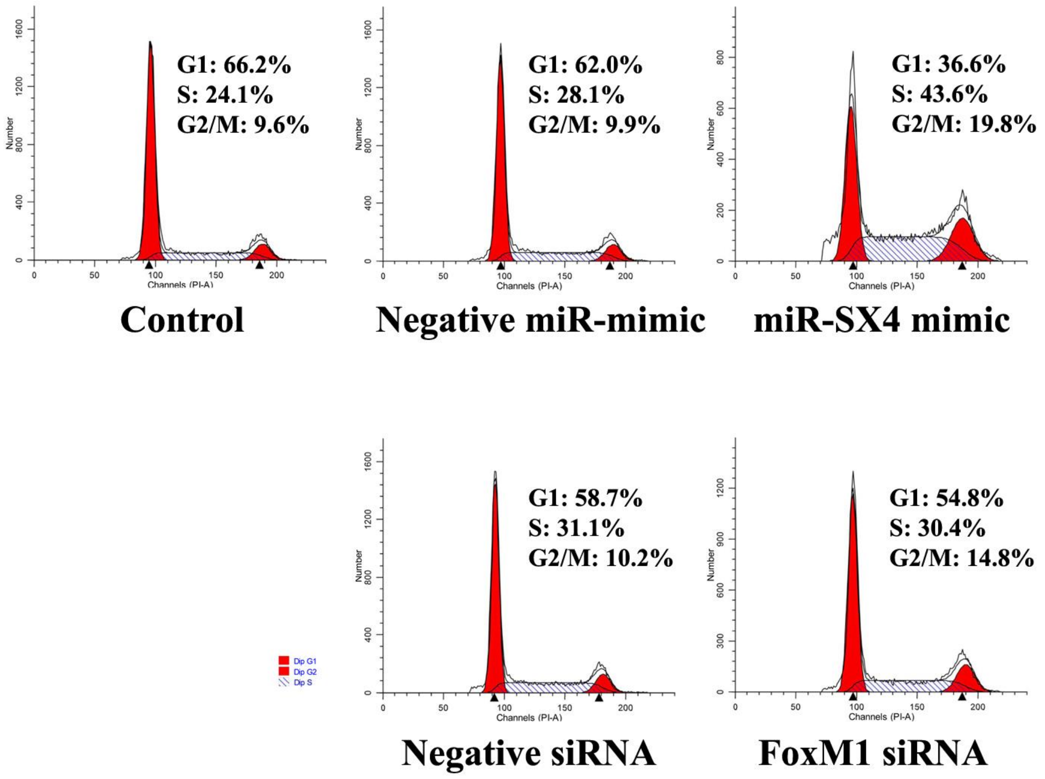

Figure S3b

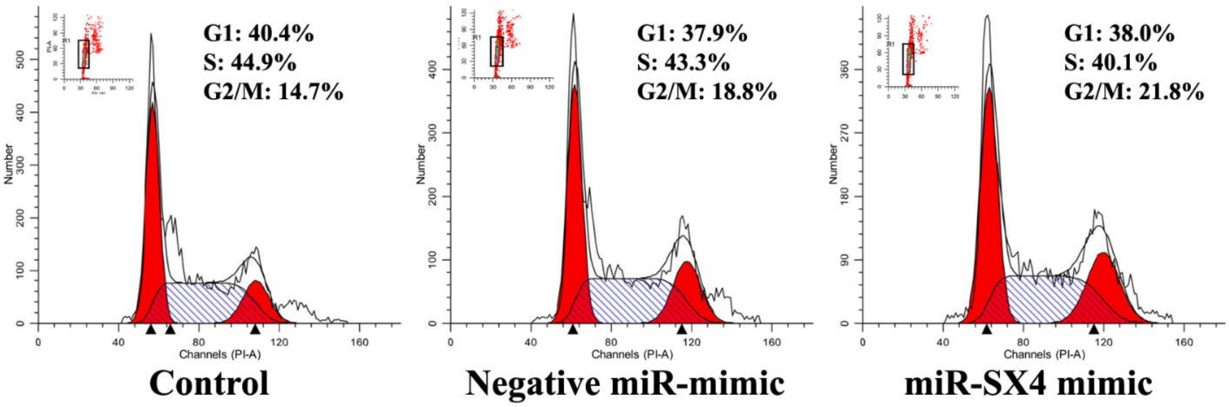

Figure S3c

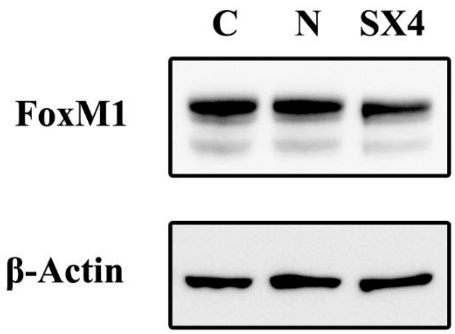

Supplementary Figure S3. (a) Histogram plot of cell cycle analysis in HeLa cells transfected with FoxM1 si-RNA (20nM, 48h). (b) Histogram plot of cell cycle analysis in HEK293T cells transfected with miR-SX4 mimic (10nM, 48h). (c) Western blot analysis of HEK293T cells transfected with miR-SX4 mimic (10nM,48h); FoxM1 and β-actin protein expression was analyzed.

Supplementary Figure S4

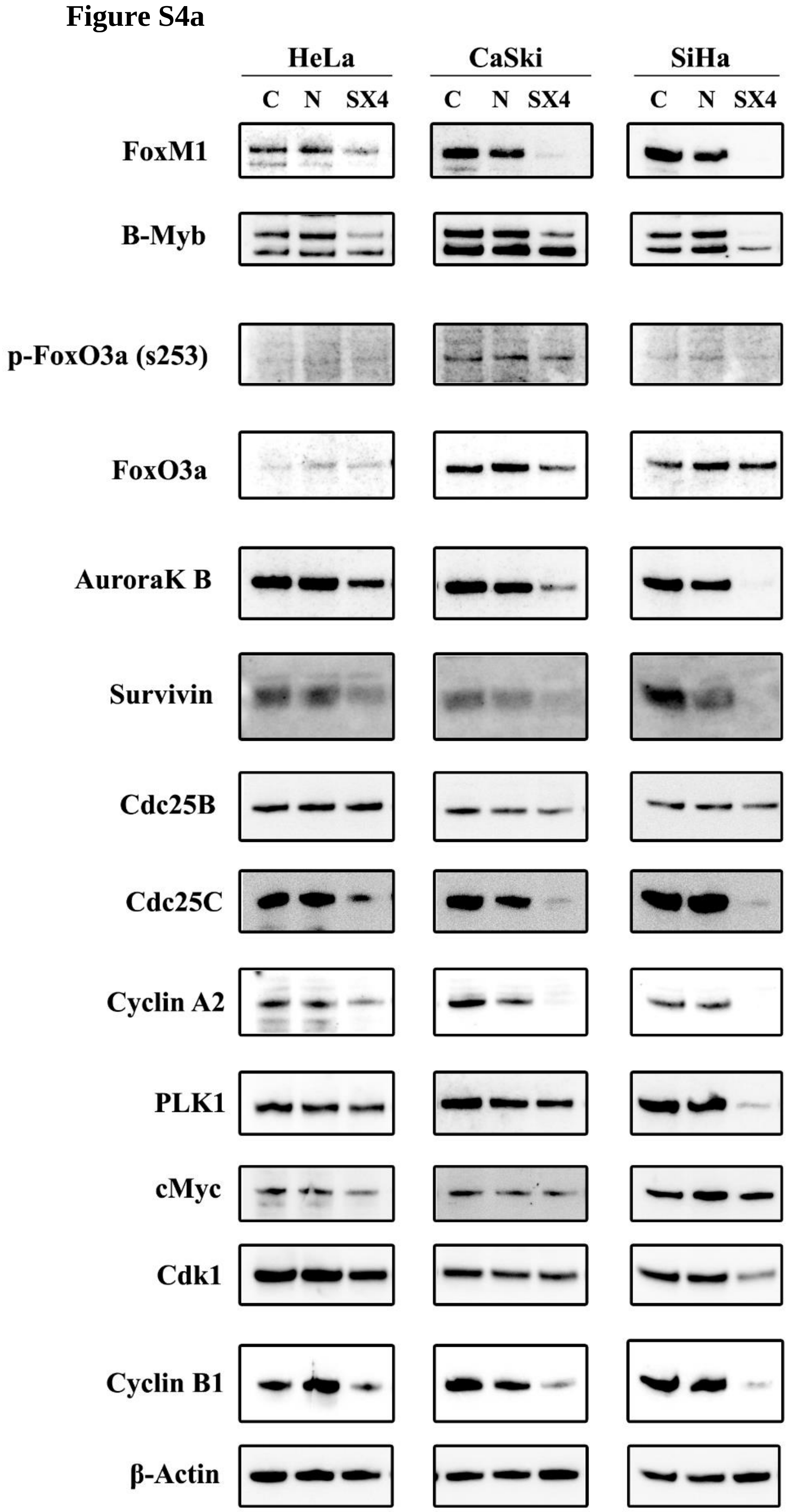

Figure S4b

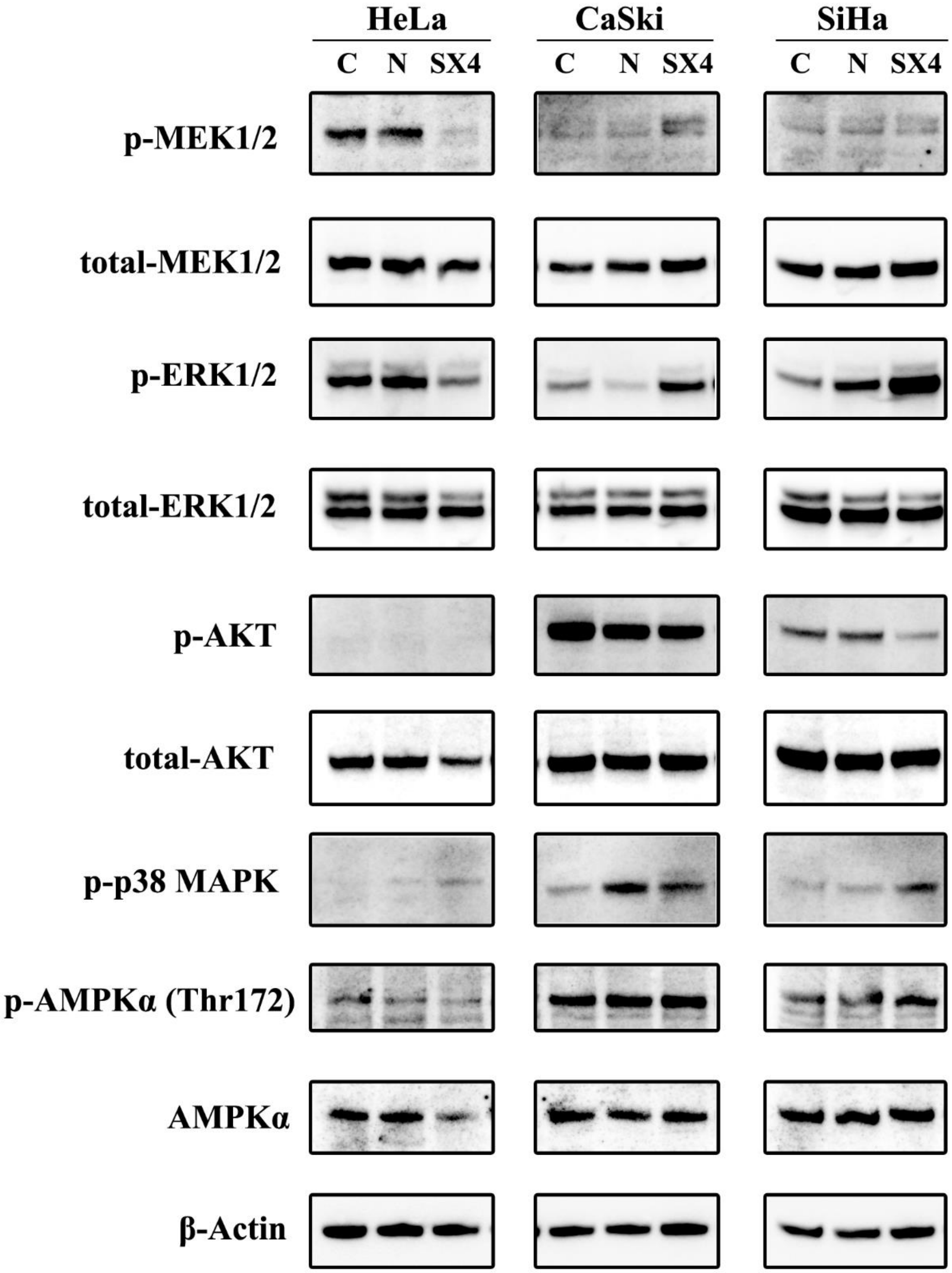

Supplementary Figure S4. HeLa, CaSki and SiHa cells untransfected (C), Negative miRNA mimic (N) and miRNA-6852 mimic (SX4) transfected cells were harvested and protein expression levels were determined by western blot for the proteins indicated.

Supplementary Figure S5

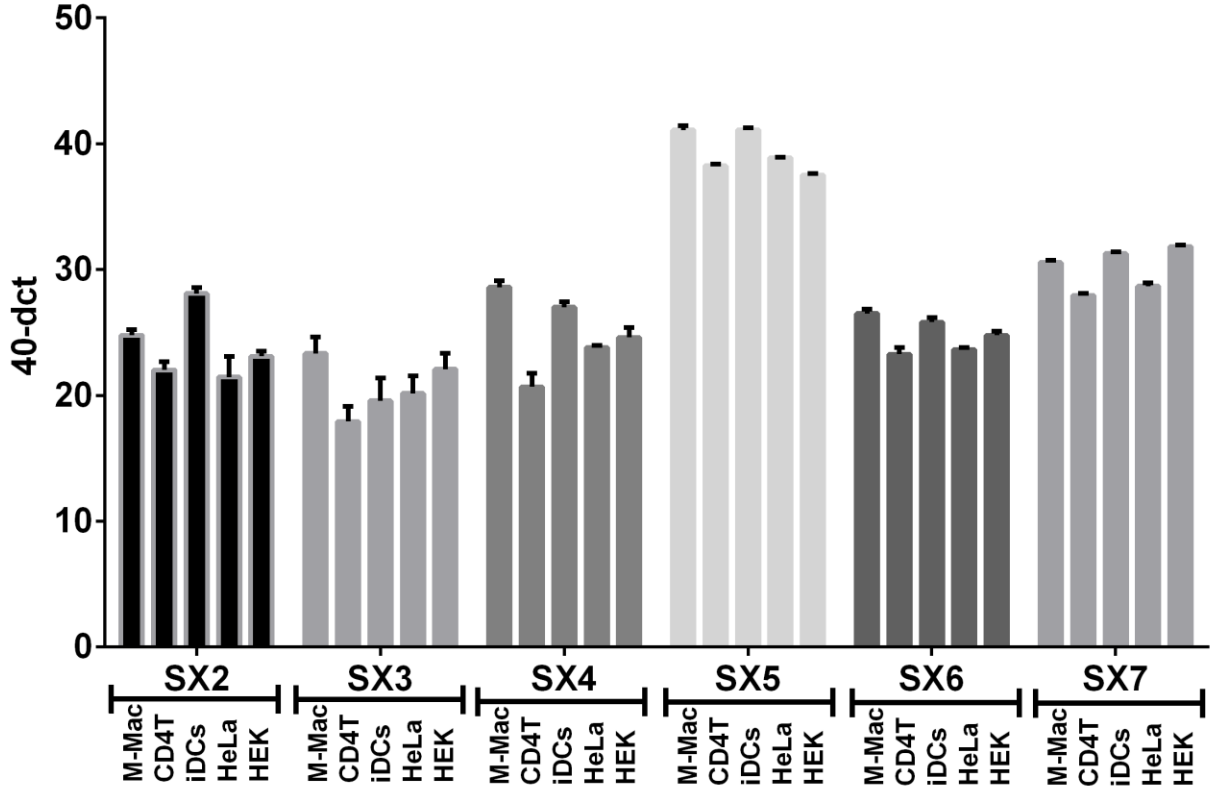

Supplementary Figure S5. Endogenous expression levels of novel miRNAs in human primary cells determined by miRNA-RT PCR.

Supplementary Figure S6

Figure S6a

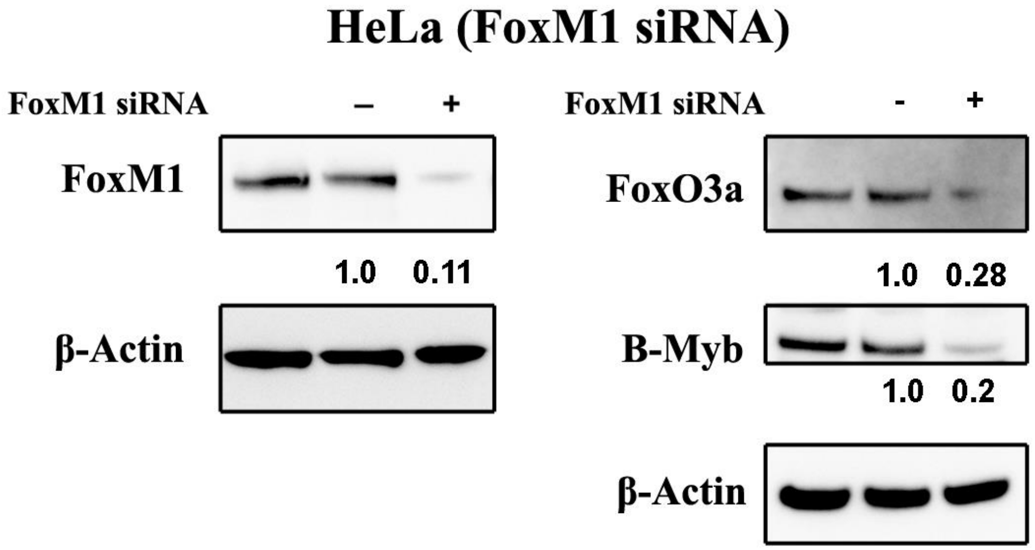

Figure S6b

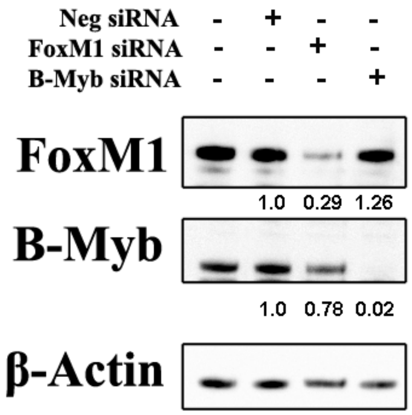

Supplementary Figure S6. (a) Western blot analysis of HeLa cells transfected with siRNA-against FoxM1 (20nM), FoxM1 protein expression (part of Figure 5c) and FoxO3a protein expression, normalized to β-actin expression. (b) Western blot analysis of HEK293 cells transfected with siRNA against FoxM1, B-myb (20nM), followed by FoxM1 and B-myb protein expression, normalized to β-actin expression. Each western blot membrane was stripped and probed with antibodies mentioned in the figure.

Supplementary Figure S7

Figure S7a

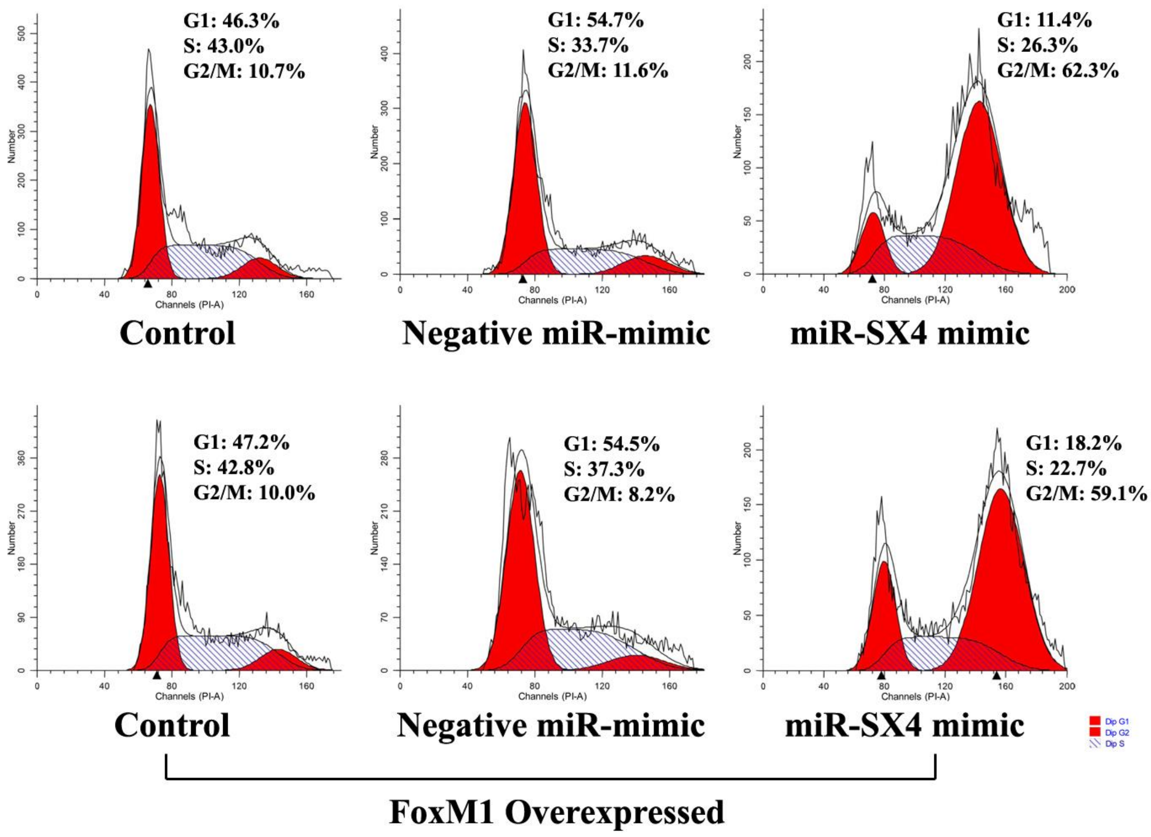

Figure S7b

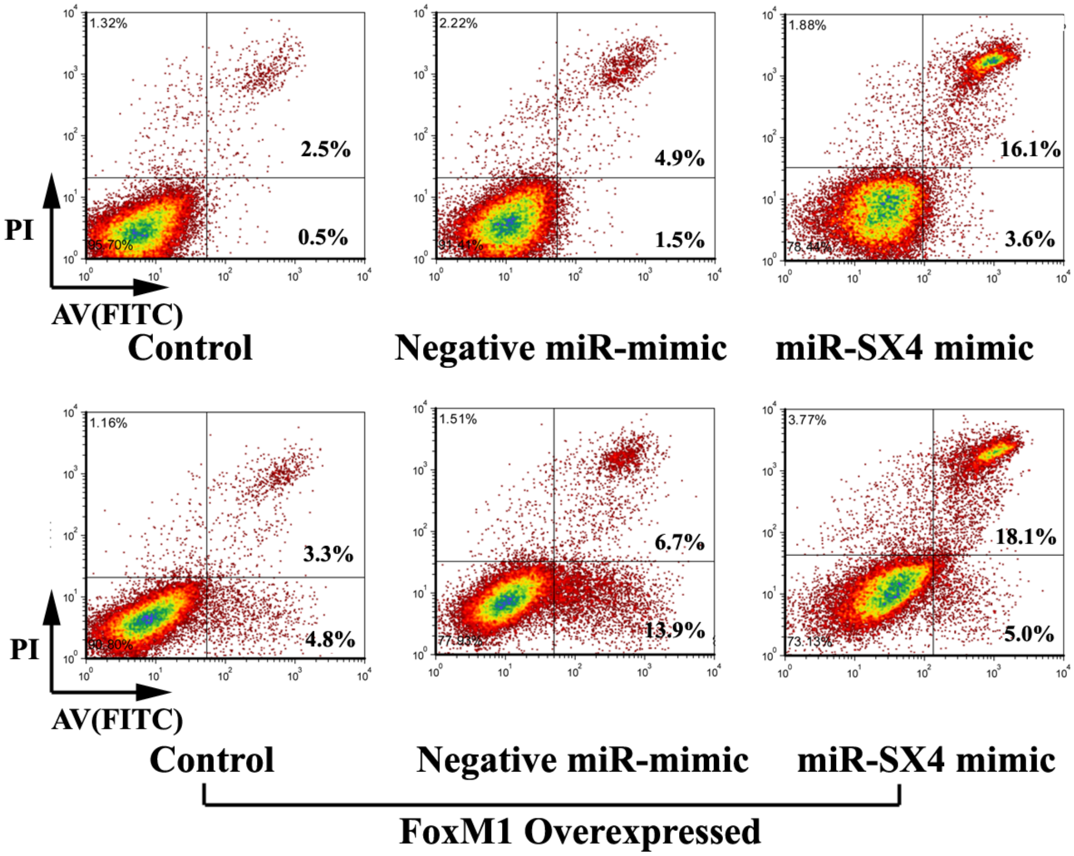
1.32%
2.5%
0.5%
PI
AV(FITC)
Control
2.22%
4.9%
1.5%
Negative miR-mimic
1.88%
16.1%
3.6%
miR-SX4 mimic
1.16%
3.3%
4.8%
PI
AV(FITC)
Control
1.51%
6.7%
13.9%
Negative miR-mimic
3.77%
18.1%
5.0%
miR-SX4 mimic
FoxM1 Overexpressed

Figure S7c

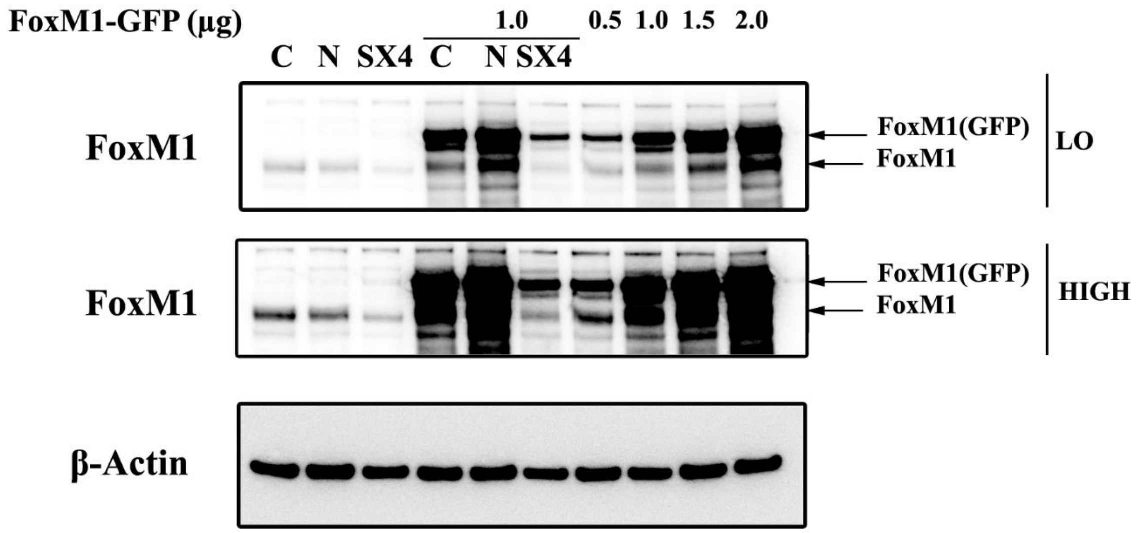

FoxM1-GFP (µg)
1.0
0.5
1.0
1.5
2.0
C
N
SX4
C
N
SX4
FoxM1
FoxM1(GFP)
FoxM1
LO
FoxM1
FoxM1(GFP)
FoxM1
HIGH
β-Actin

Figure S7d

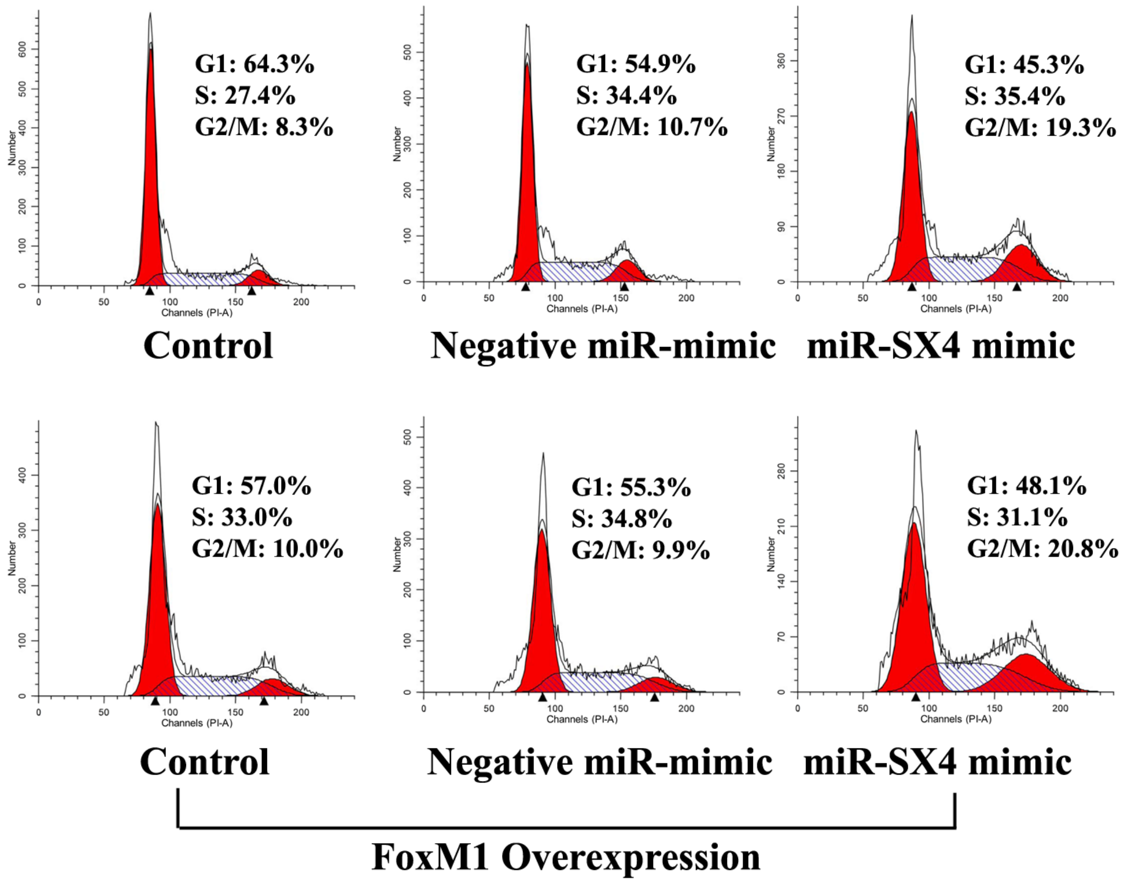
G1: 64.3%
S: 27.4%
G2/M: 8.3%
G1: 54.9%
S: 34.4%
G2/M: 10.7%
G1: 45.3%
S: 35.4%
G2/M: 19.3%
Number
Channels (PI-A)
Control
Negative miR-mimic
miR-SX4 mimic
G1: 57.0%
S: 33.0%
G2/M: 10.0%
G1: 55.3%
S: 34.8%
G2/M: 9.9%
G1: 48.1%
S: 31.1%
G2/M: 20.8%
Control
Negative miR-mimic
miR-SX4 mimic
FoxM1 Overexpression

Figure S7e

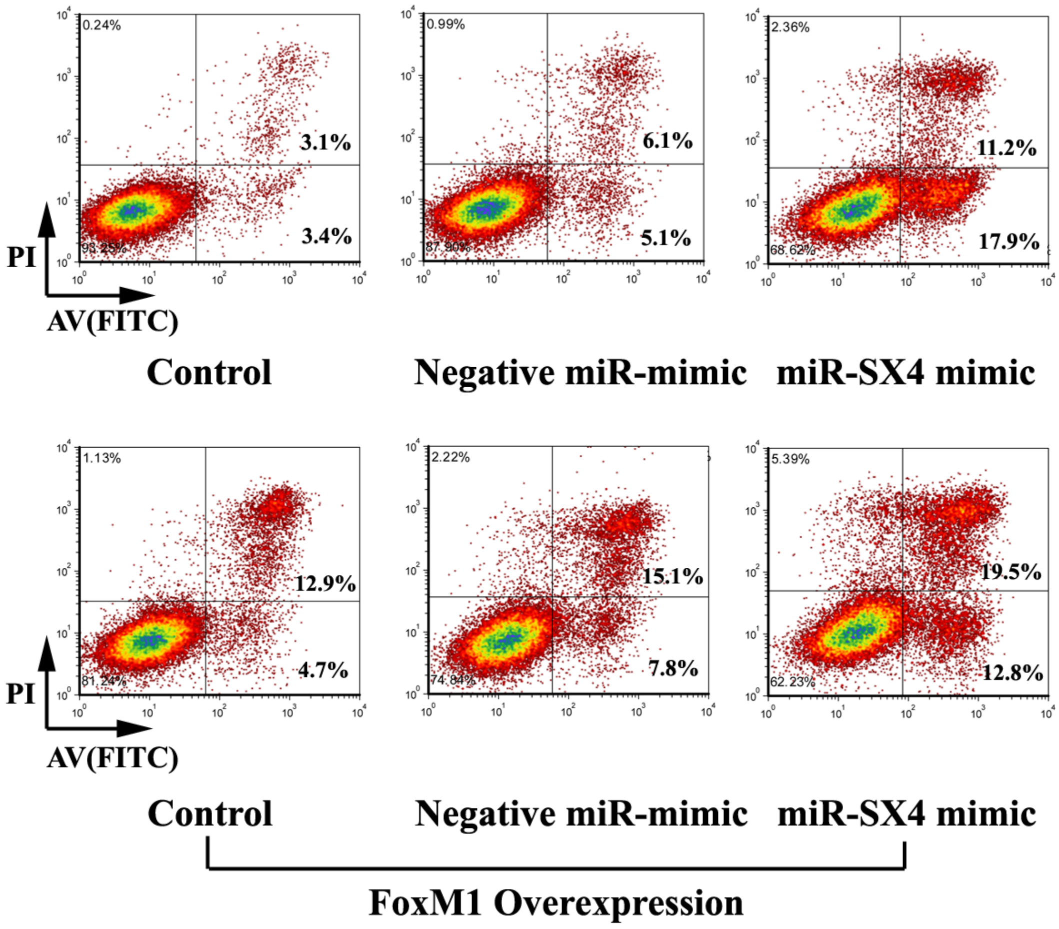
0.24%
3.1%
3.4%
98.26%
PI
AV(FITC)
Control
0.99%
6.1%
5.1%
87.30%
Negative miR-mimic
2.36%
11.2%
17.9%
68.62%
miR-SX4 mimic
1.13%
12.9%
4.7%
81.24%
PI
AV(FITC)
Control
2.22%
15.1%
7.8%
74.84%
Negative miR-mimic
5.39%
19.5%
12.8%
62.23%
miR-SX4 mimic
FoxM1 Overexpression

Figure S7c

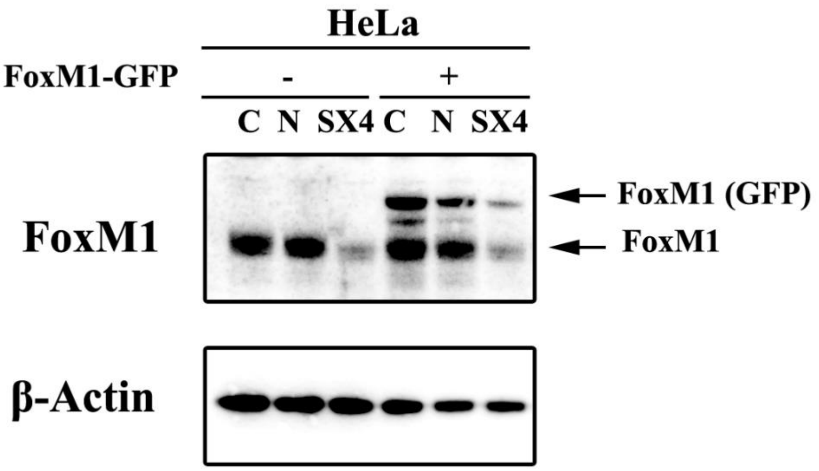

Supplementary Figure S7. (a) Histogram plot of cell cycle analysis in HEK293 cells transfected with miR-SX4 (10nM); 24h post-transfection plasmid DNA expressing FoxM1-GFP (1µg) was transfected for another 48h in the indicated cells (lower panel). (b) Dot plot of apoptosis/necrosis analysis in HEK293 cells transfected with miR-SX4 (10nM); 24h post-transfection plasmid DNA expressing FoxM1-GFP (1µg) was transfected for another 48h in the indicated cells (lower panel). (c) Western blot analysis of the same sets of HEK293 cells to confirm the overexpression of FoxM1 protein (Lo is 1 sec and High is 1 min exposure). (d) Histogram plot of cell cycle analysis in HeLa cells transfected with plasmid DNA expressing FoxM1-GFP (1µg); 24h post-transfection miR-SX4 or negative miR-mimic (10nM) was transfected for another 48h in the indicated cells (lower panel). (e) Dot plot of apoptosis/necrosis analysis in HeLa cells transfected with plasmid DNA expressing FoxM1-GFP (1µg); 24h post-transfection miR-SX4 or negative miR-mimic (10nM) was transfected for another 48h in the indicated cells (lower panel). GFP+ cells were selected for cell cycle and apoptosis/necrosis analysis. (f) Western blot analysis of the same sets of HeLa cells to confirm the overexpression of FoxM1 protein.

Supplementary Figure S8

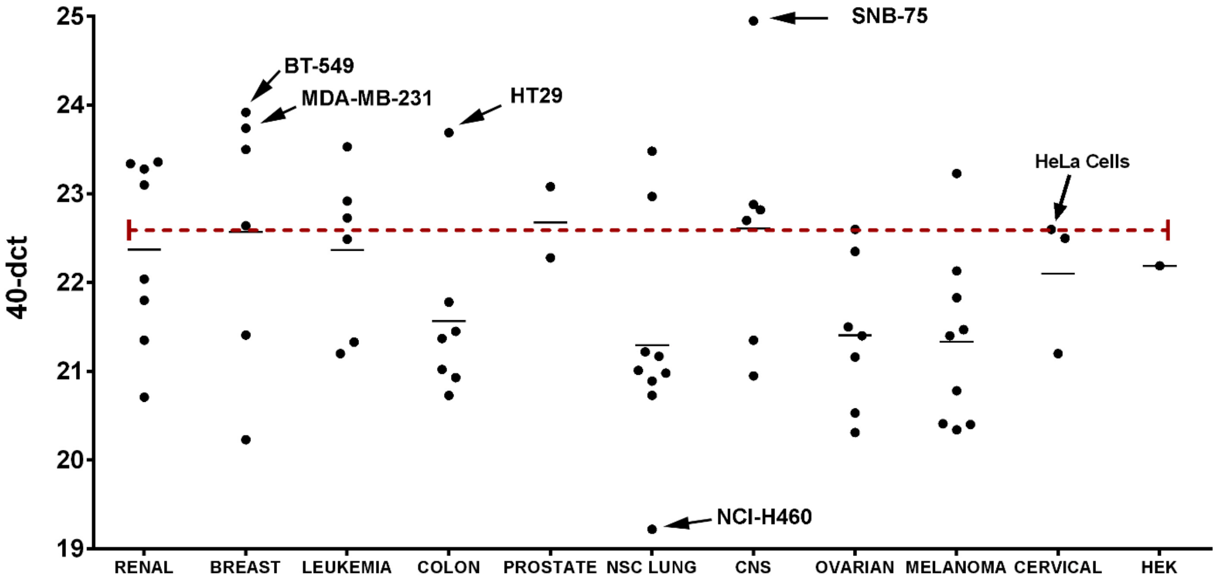

Supplementary Figure S8. Endogenous miRNA-SX4 levels in NCI-60 panel of human tumor cell lines. Endogenous miR-SX4 expression levels in NCI-60 panel of human tumor cell lines including cervical cancer cell line (HeLa, SiHa and CaSki) and HEK293 cells. Y-axis represents the (40-Δdct) value of miR-SX4 normalized to RNU44 internal control. X-axis represents the different panel of tumor cells based on the tissue of origin.

Supplementary Figure S9

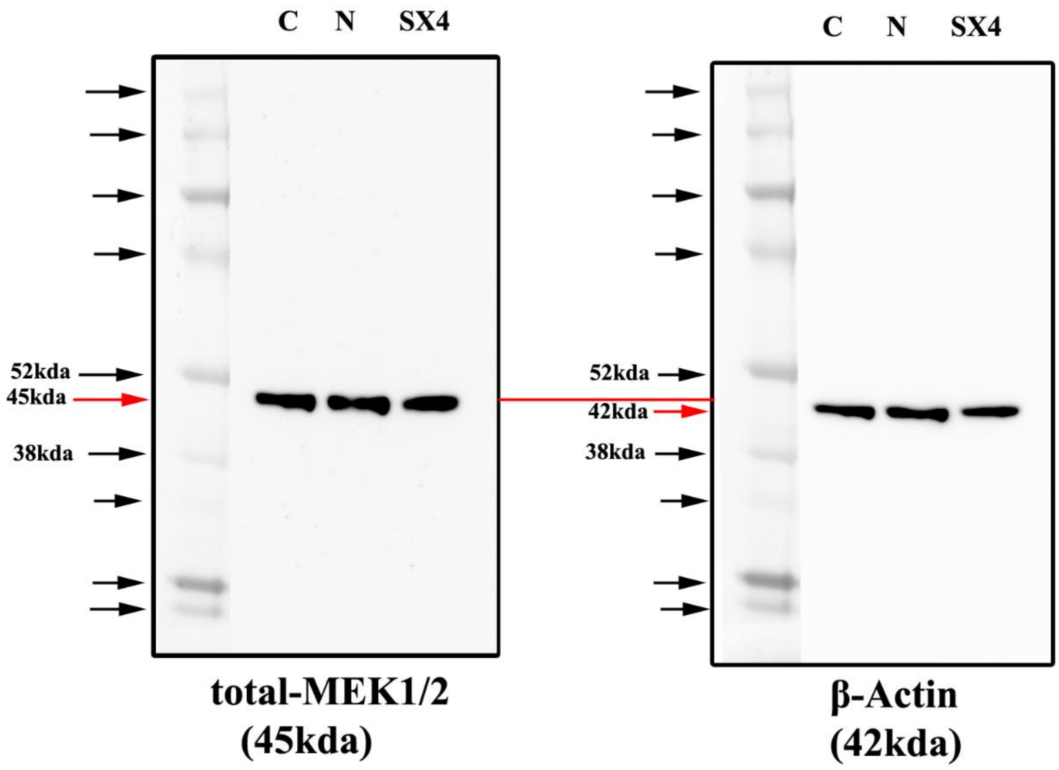

Supplementary Figure S9. Western Blot analysis of HEK293 cells transfected with miR-SX4. The same figure with the indicated bands were cut and shown in figure 4b,i. The above figure is the entire picture of the western blot indicating total-MEK1/2 band was detected around 45kda and β-Actin band was detected around 42kda.

Supplementary Figure S10

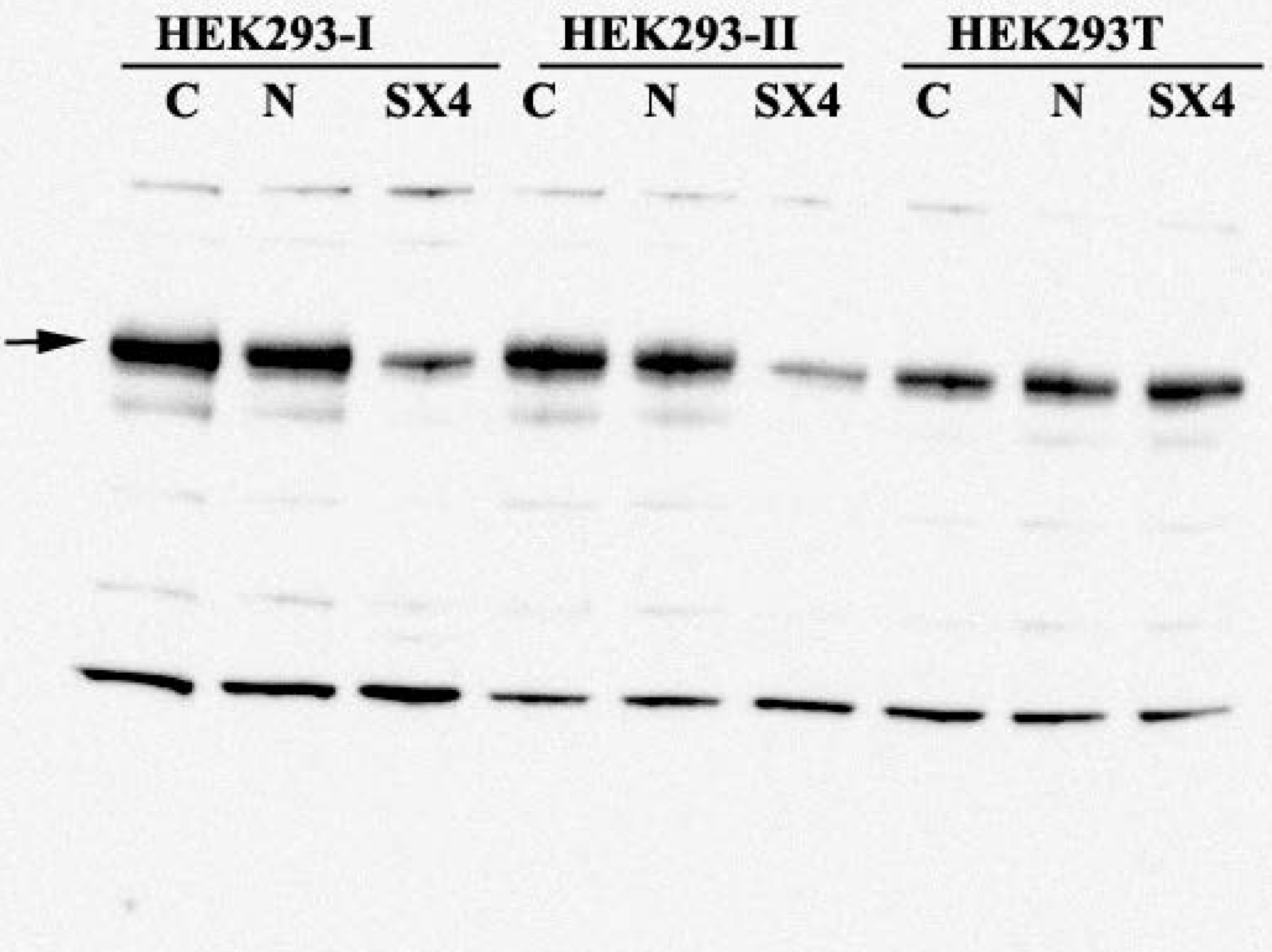

Figure 2c- FoxM1

Figure 2c β-Actin

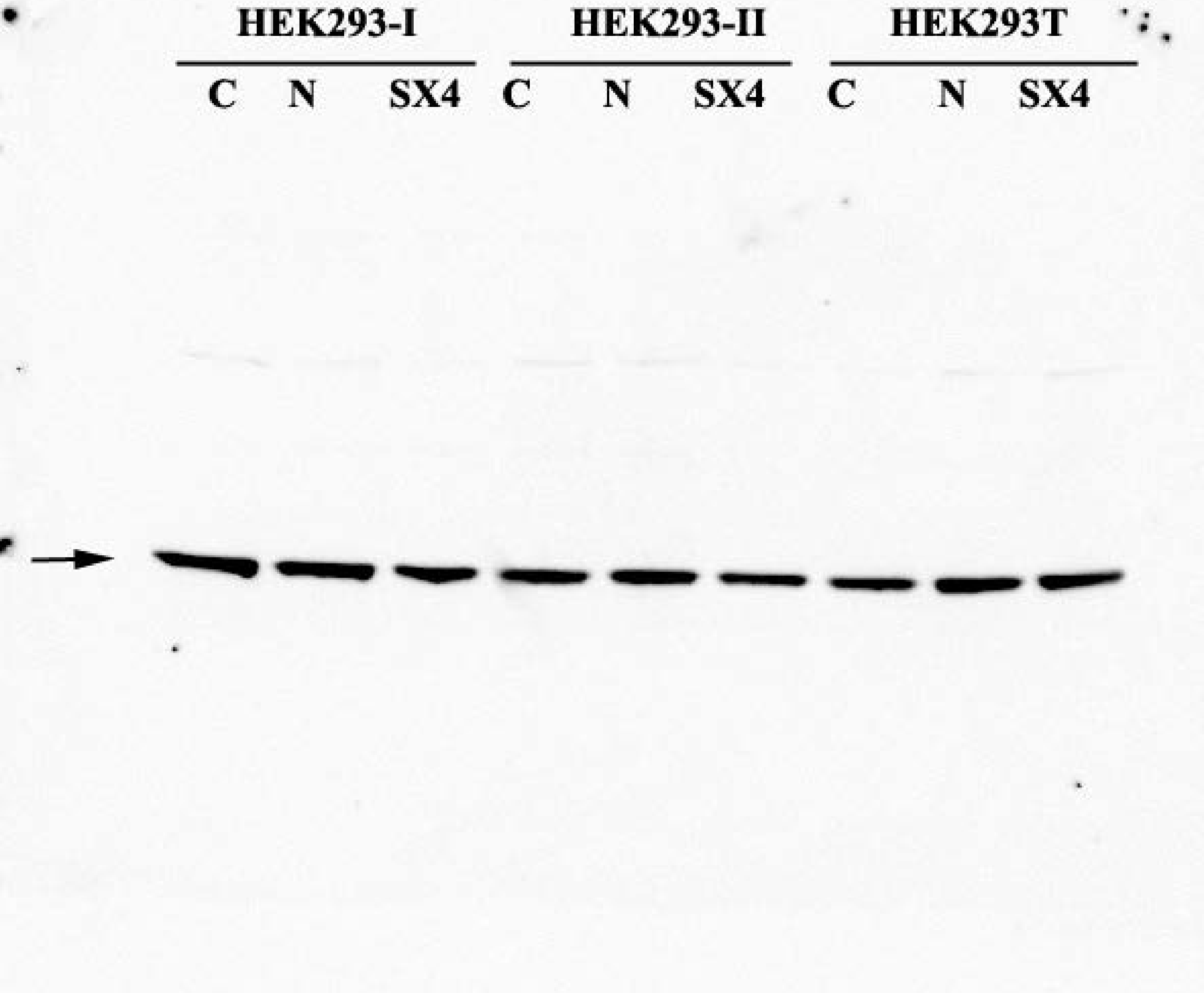

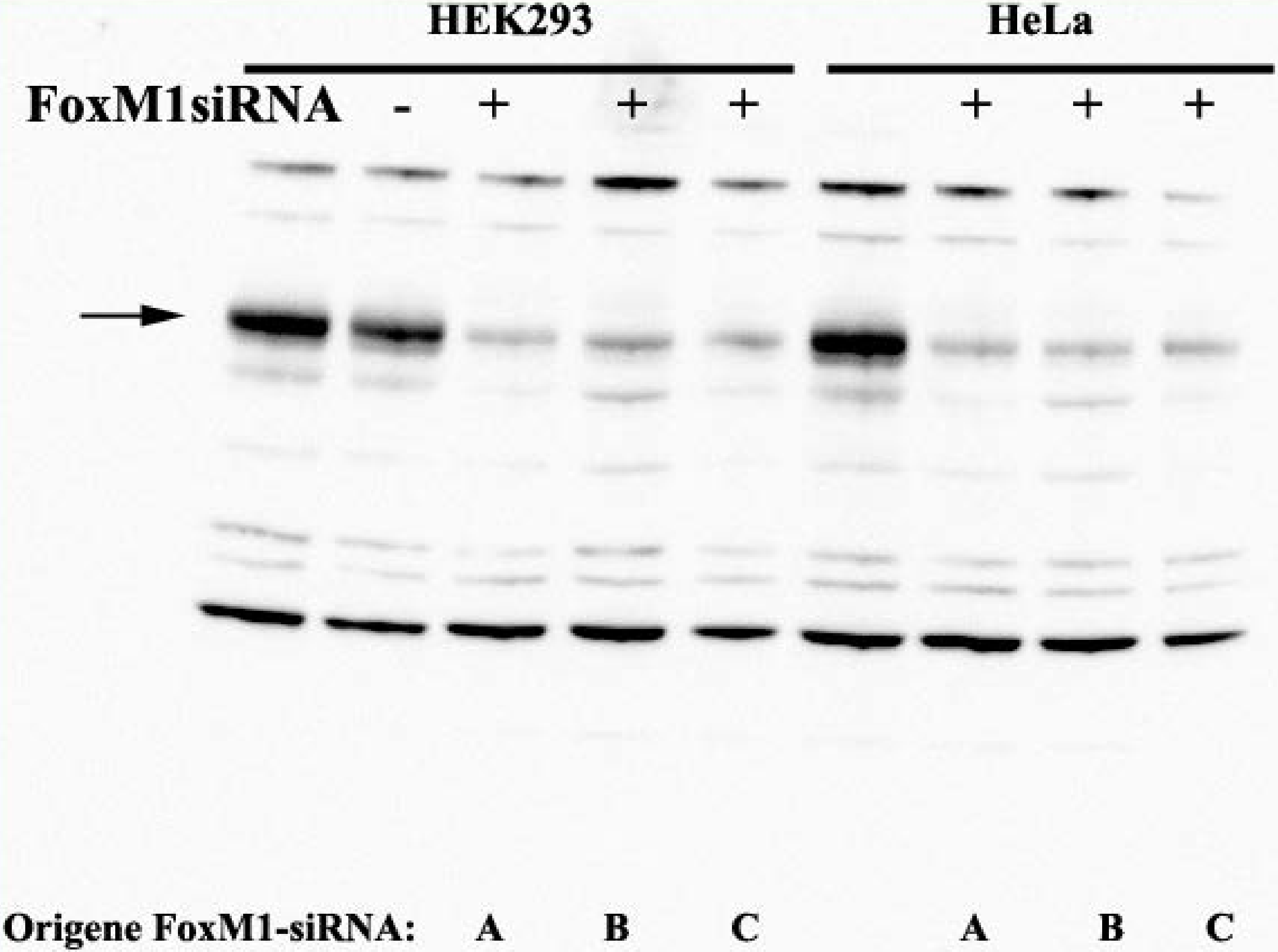

Origene FoxM1-siRNA (A) chosen for futher studies

Figure 3a: FoxM1

Figure 3a: β-Actin

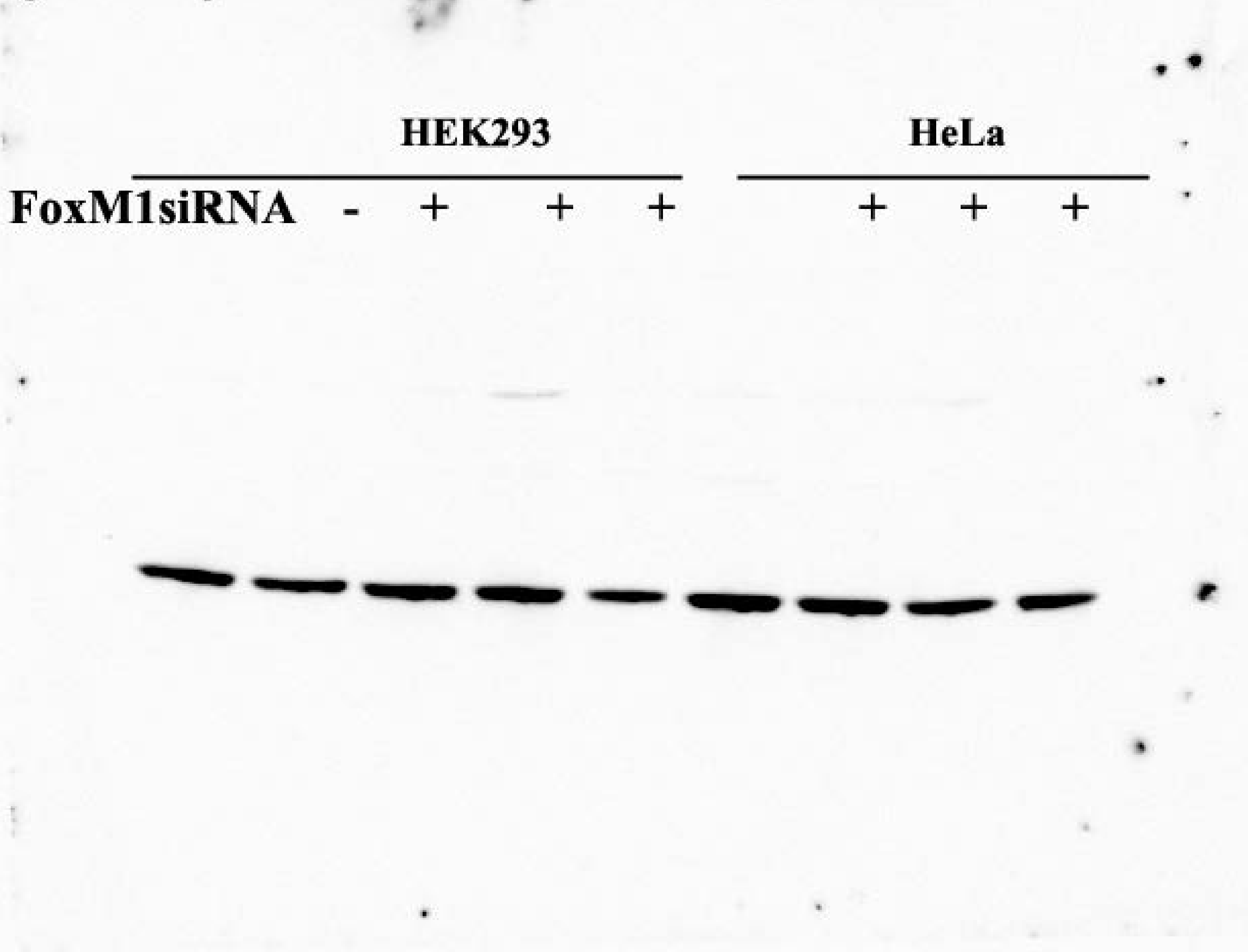

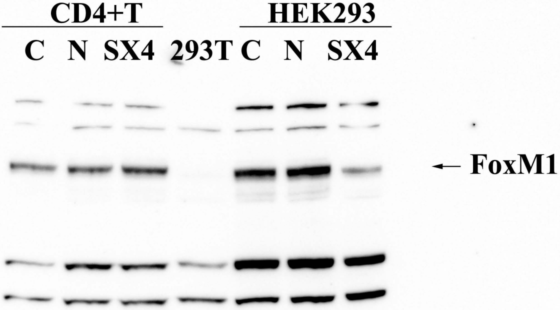

Figure 4a-i: FoxM1

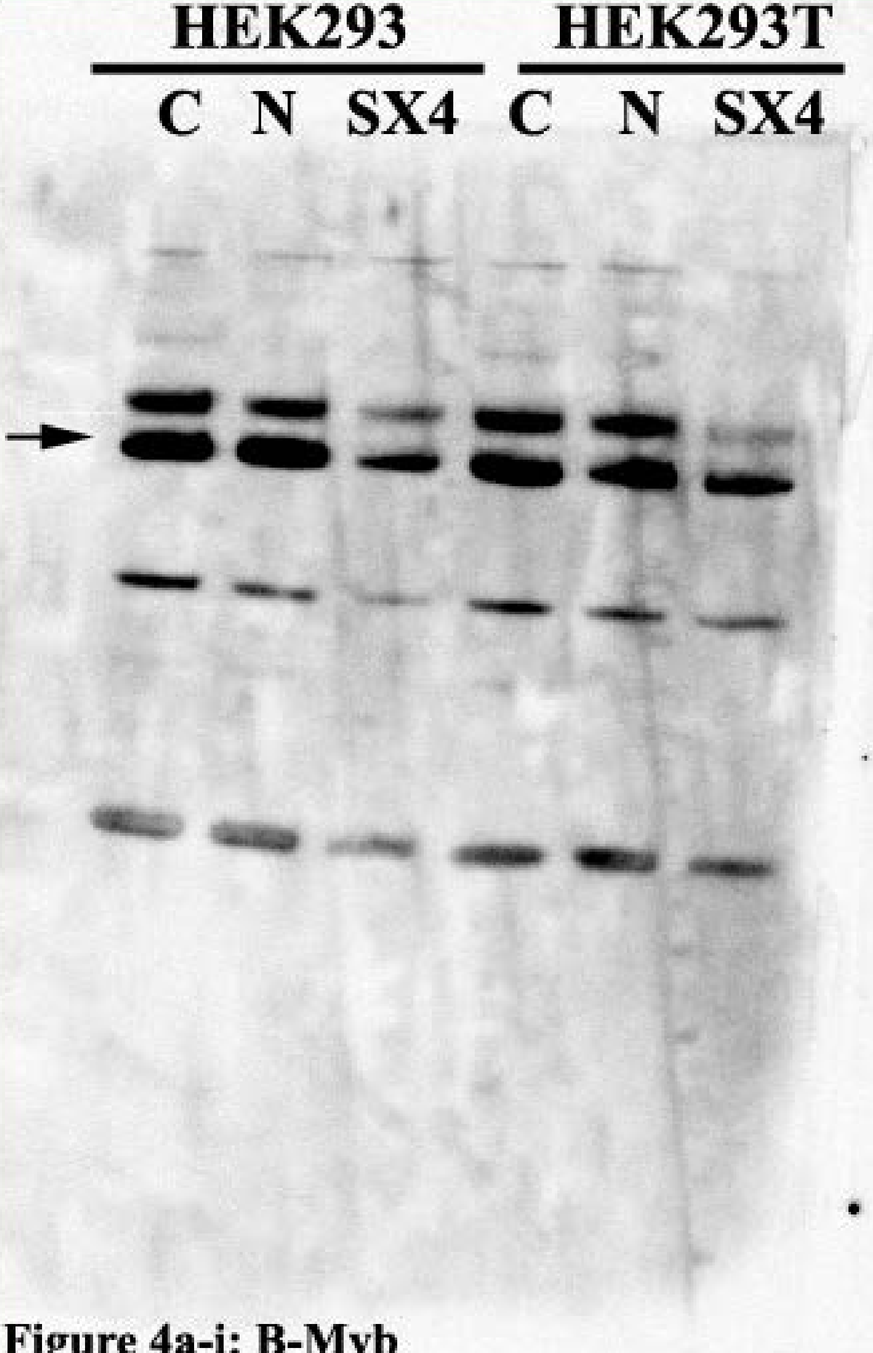

Figure 4a-i: B-Myb

Figure 4a-i-β-Actin

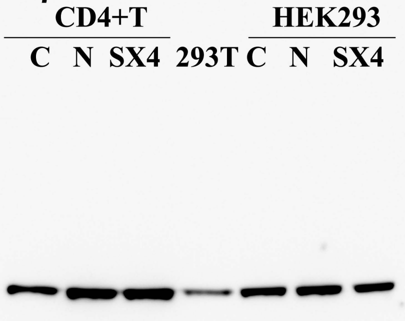

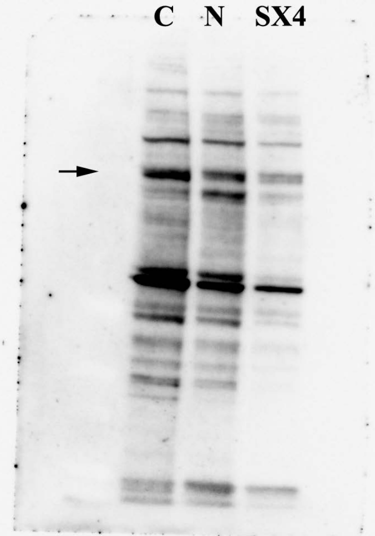

Figure 4a-ii: p-FoxO3a (s253)

Figure 4a-ii: FoxO3a

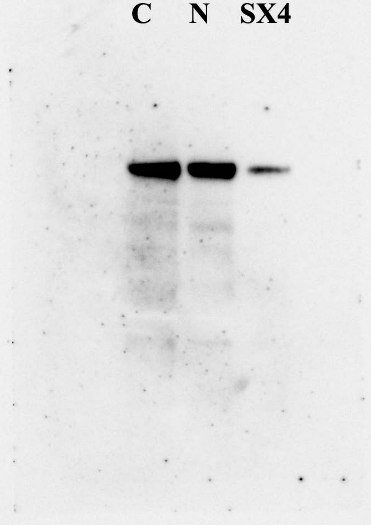

Figure 4a-ii: β-Actin

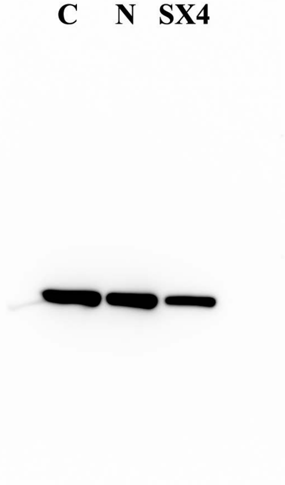

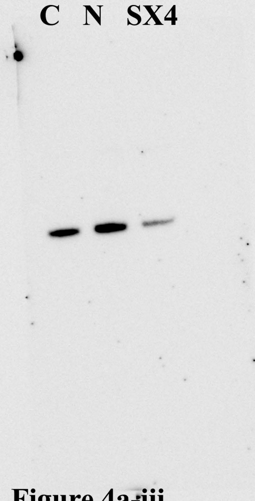

Figure 4a-iii
AuroraK B

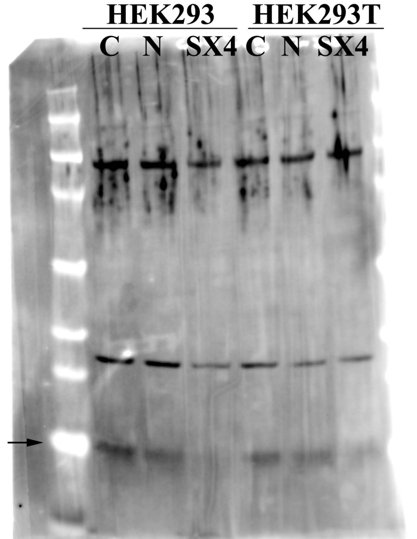

Figure 4a-iii: Survivin

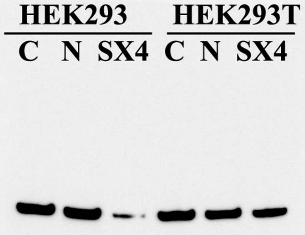

Figure 4a-iii: CDC25B

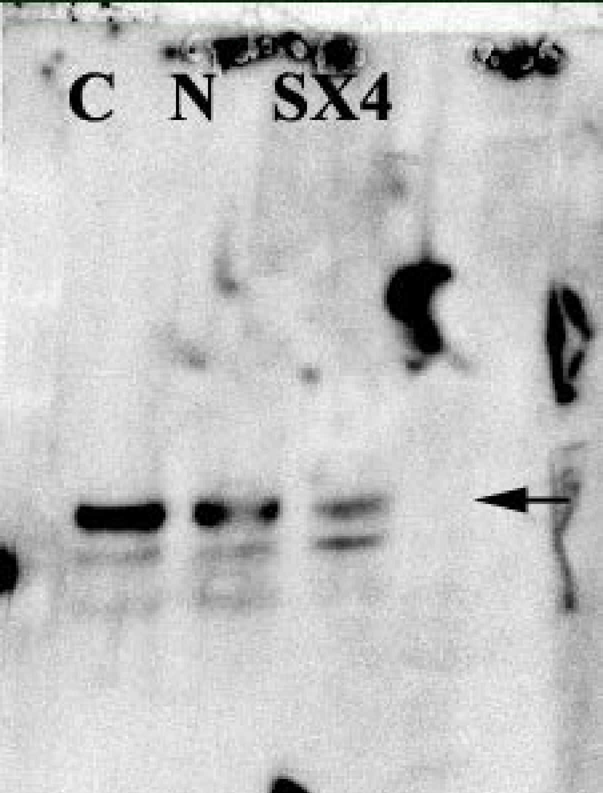

Figure 4a-iii
CDC25C

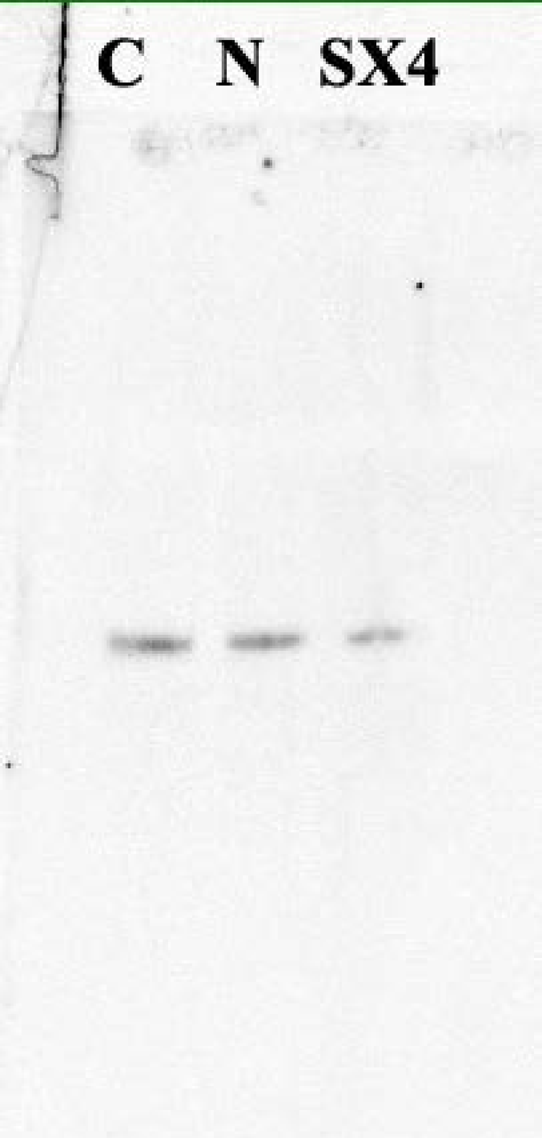

Figure 4a-iii
Cyclin A2

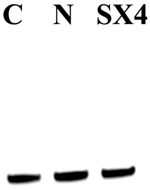

Figure 4a-iii: PLK1

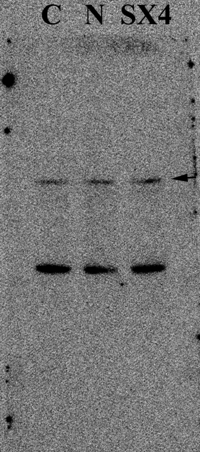

Figure 4a-iii
Myc

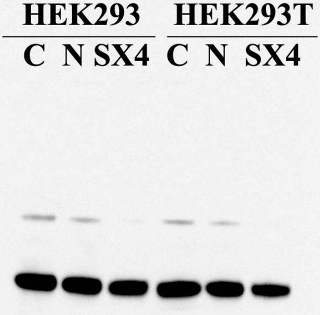

Figure 4a-iii: Cdk1

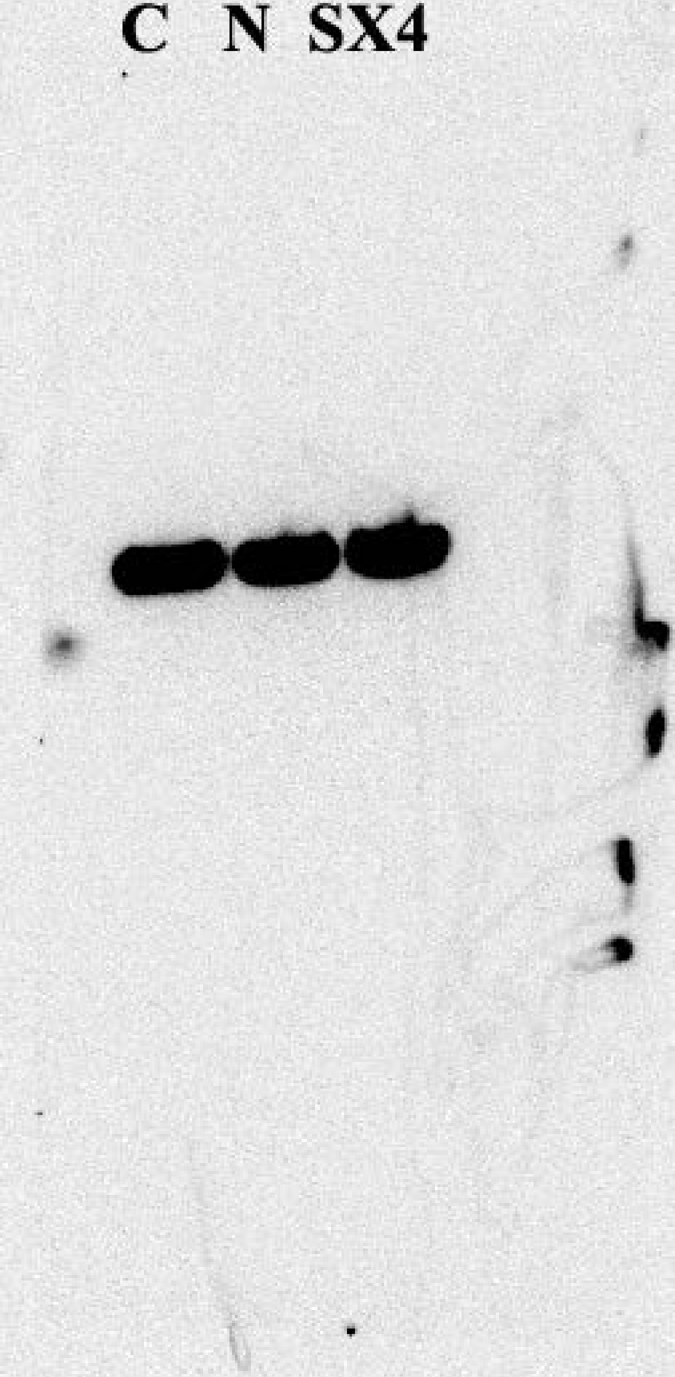

Figure 4a-iii: CyclinB1

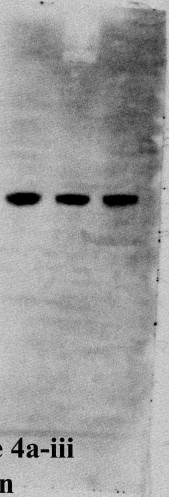

Figure 4a-iii
β-Actin

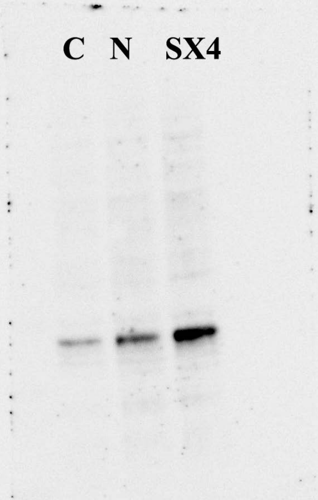

Figure 4b-i: pMEK1/2

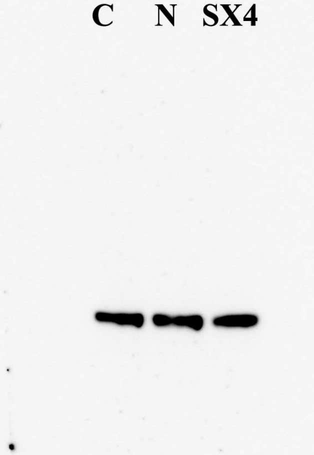

Figure 4b-i: total MEK1/2

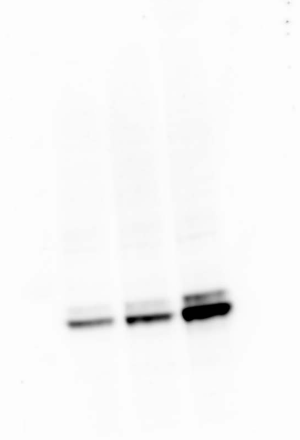

Figure 4b-i: pERK1/2

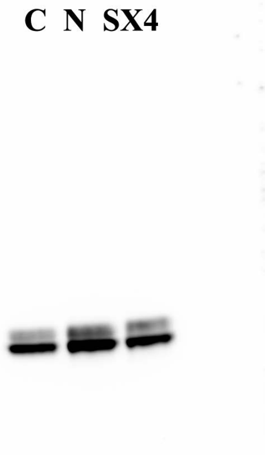

Figure 4b-i: total ERK1/2

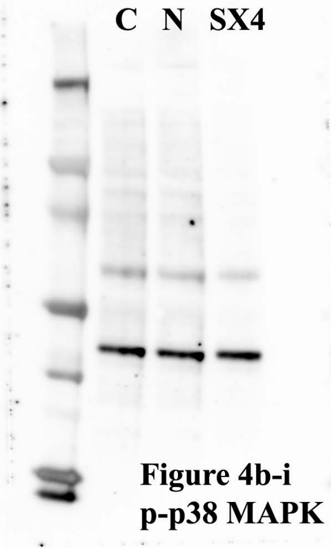

Figure 4b-i
p-p38 MAPK

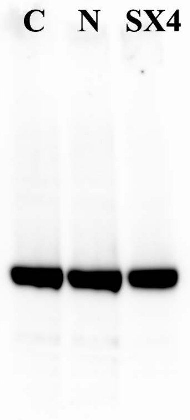

Figure 4b-i: p-AMPKα

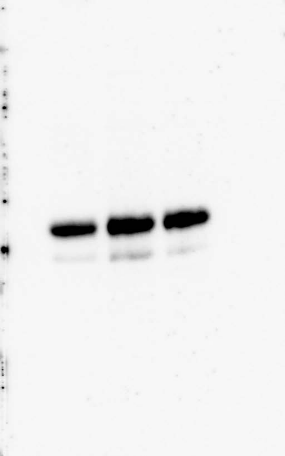

Figure 4b-i
total AMPKα

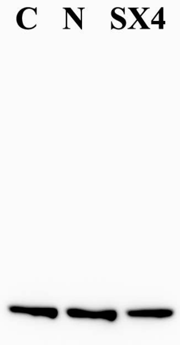

Figure 4b-i: β-Actin

Figure 4bii p-AKT

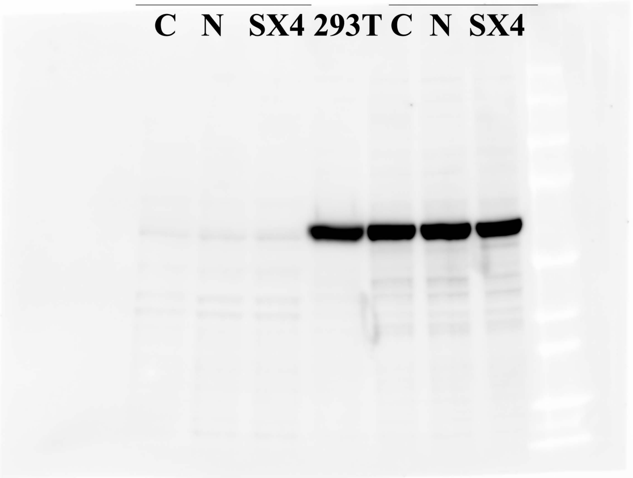

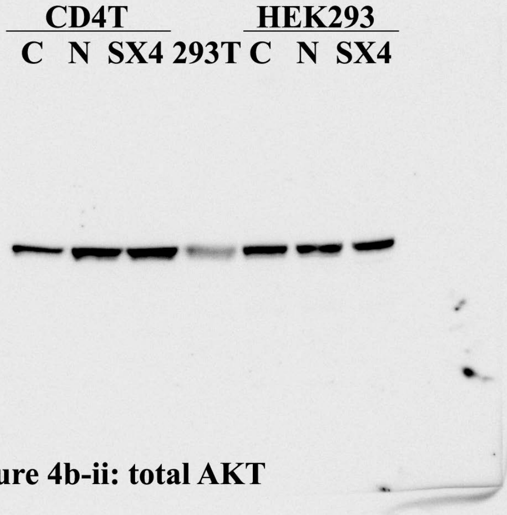

Figure 4b-ii: total AKT

Figure 4bii-β-Actin

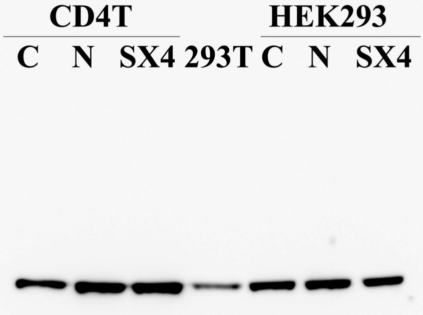

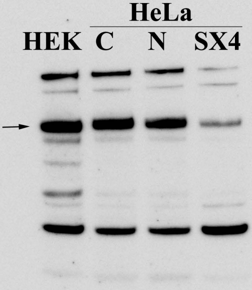

Figure 5b: HeLa (FoxM1)

Figure 5b: β-Actin

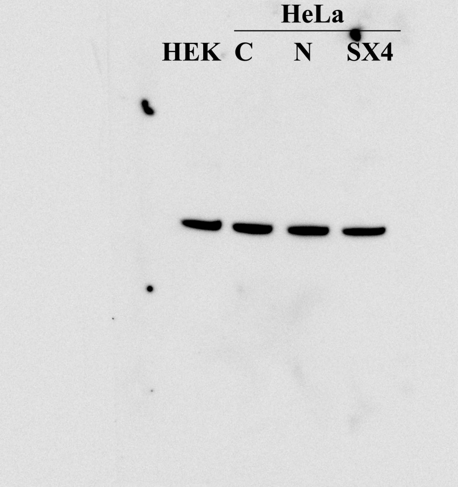

Figure 5b: FoxM1

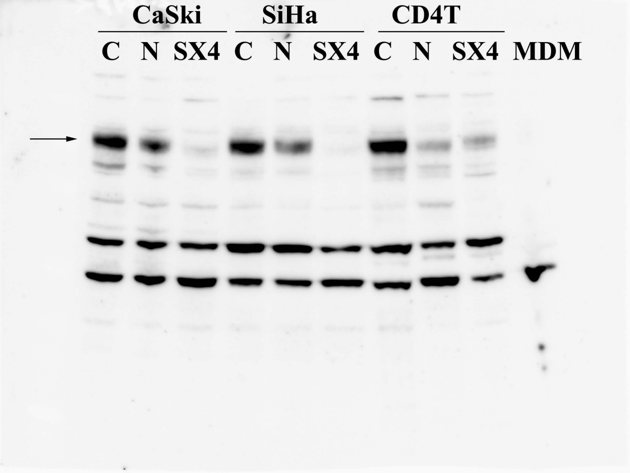

Figure 5b: β-Actin

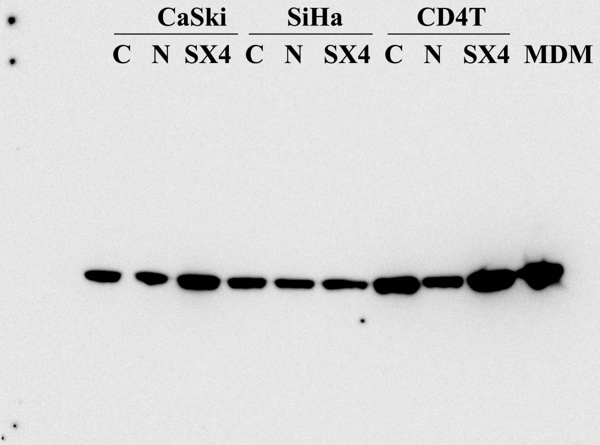

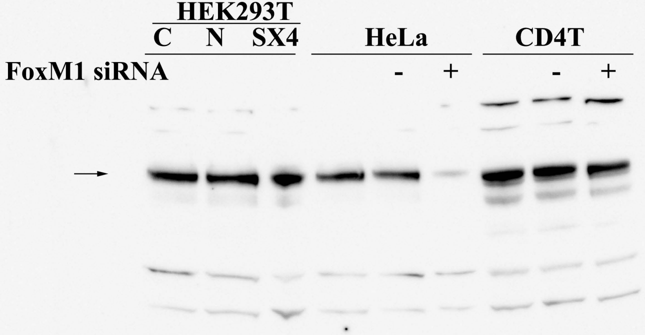

Figure 5c: FoxM1

Figure 5c: Hela:β-Actin

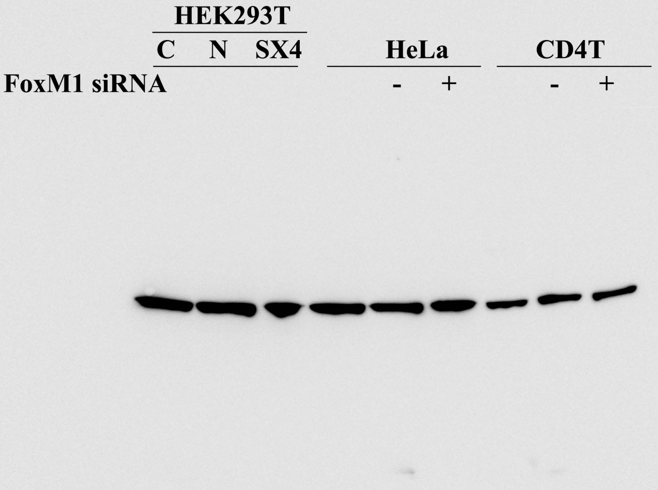

Figure 5c: FoxM1

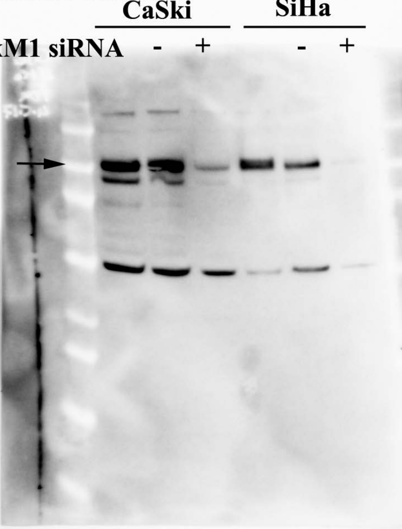

Figure 5c: β-Actin (CaSki)

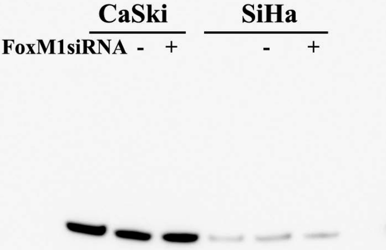

Figure 5c: β-Actin (SiHa)

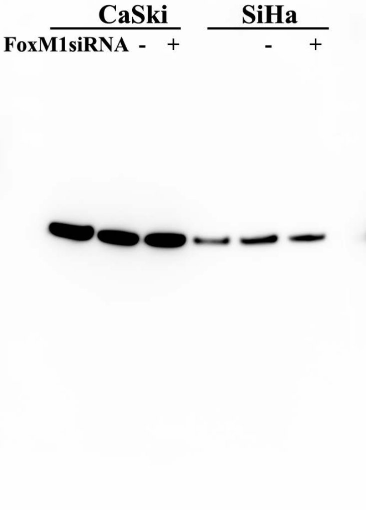

Supplement: Supplementary file 1 — Supplementary Figures [file 41598_2018_19259_MOESM1_ESM.pdf]
